# Significant Effects of Maternal Diet During Pregnancy on the Murine Fetal Brain Transcriptome and Offspring Behavior

**DOI:** 10.3389/fnins.2019.01335

**Published:** 2019-12-17

**Authors:** Andrea G. Edlow, Faycal Guedj, Deanna Sverdlov, Jeroen L. A. Pennings, Diana W. Bianchi

**Affiliations:** ^1^Mother Infant Research Institute, Tufts Medical Center, Boston, MA, United States; ^2^Department of Obstetrics and Gynecology, Tufts Medical Center, Boston, MA, United States; ^3^National Institute for Public Health and the Environment, Bilthoven, Netherlands

**Keywords:** maternal diet, fetal brain, transcriptome, strength, coordination, sensory, micronutrient, fatty acid

## Abstract

**Background:**

Maternal over- and undernutrition in pregnancy plays a critical role in fetal brain development and function. The effects of different maternal diet compositions on intrauterine programing of the fetal brain is a lesser-explored area. The goal of this study was to investigate the impact of two chowmaternal diets on fetal brain gene expression signatures, fetal/neonatal growth, and neonatal and adult behavior in a mouse model.

**Methods:**

Throughout pregnancy and lactation, female C57Bl/6J mice were fed one of two standard, commercially available chow diets (pellet versus powder). The powdered chow diet was relatively deficient in micronutrients and enriched for carbohydrates and n-3 long-chain polyunsaturated fatty acids compared to the pelleted chow. RNA was extracted from embryonic day 15.5 forebrains and hybridized to whole genome expression microarrays (*N* = 5/maternal diet group). Functional analyses of significantly differentially expressed fetal brain genes were performed using Ingenuity Pathways Analysis and Gene Set Enrichment Analysis. Neonatal behavior was assessed using a validated scale (*N* = 62 pellet-exposed and 31 powder-exposed). Hippocampal learning, locomotor behavior, and motor coordination were assessed in a subset of adults using fear conditioning, open field testing, and Rotarod tests (*N* = 16 pellet-exposed, 14 powder-exposed).

**Results:**

Comparing powdered to pelleted chow diets, neither maternal weight trajectory in pregnancy nor embryo size differed. Maternal powdered chow diet was associated with 1647 differentially expressed fetal brain genes. Functional analyses identified significant upregulation of canonical pathways and upstream regulators involved in cell cycle regulation, synaptic plasticity, and sensory nervous system development in the fetal brain, and significant downregulation of pathways related to cell and embryo death. Pathways related to DNA damage response, brain immune response, amino acid and fatty acid transport, and dopaminergic signaling were significantly dysregulated. Powdered chow-exposed neonates were significantly longer but not heavier than pelleted chow-exposed counterparts. On neonatal behavioral testing, powdered chow-exposed neonates achieved coordination- and strength-related milestones significantly earlier, but sensory maturation reflexes significantly later. On adult behavioral testing, powdered chow-exposed offspring exhibited hyperactivity and hippocampal learning deficits.

**Conclusion:**

In wild-type offspring, two diets that differed primarily with respect to micronutrient composition had significant effects on the fetal brain transcriptome, neonatal and adult behavior. These effects did not appear to be mediated by alterations in gross maternal nutritional status nor fetal/neonatal weight. Maternal dietary content is an important variable to consider for investigators evaluating fetal brain development and offspring behavior.

## Introduction

In both human epidemiologic and animal model studies, maternal under- and overnutrition in pregnancy has been well-demonstrated to have a deleterious impact on fetal brain development and offspring behavior (Tozuka et al., 2009, 2010; White et al., 2009; Antonow-Schlorke et al., 2011; Brion et al., 2011; Krakowiak et al., 2012; Kang et al., 2014; Edlow et al., 2016a, b; Li et al., 2016; van der Burg et al., 2016; Veena et al., 2016; Edlow, 2017; Winther et al., 2018). The impact of maternal dietary micronutrient composition on the developing fetal brain has not been as well-characterized. Dietary micronutrient deficiency may have broader relevance to conditions such as maternal obesity, in which a relative micronutrient deficiency has been proposed due to poor maternal dietary quality (Pinhas-Hamiel et al., 2003; Kimmons et al., 2006; Laraia et al., 2007; Darnton-Hill and Mkparu, 2015; Jones et al., 2016; Scholing et al., 2018). The relative contribution of maternal micronutrient deficiency compared to maternal pre-pregnancy obesity or maternal high-fat diet in mediating some of the deleterious effects of maternal obesity on the developing brain is unknown. Similarly, to what extent the deleterious impact of maternal undernutrition in pregnancy on the developing fetal brain may be mediated by micronutrient versus macronutrient deficiency in the diet remains to be elucidated.

While the impact of maternal macronutrient and micronutrient intake in pregnancy has been examined in several human observational studies, these focus primarily on the impact of maternal intake on pregnancy outcomes such as preeclampsia and preterm birth, on fetal growth trajectory, and on neonatal outcomes such as small- and large-for-gestational age, and incidence of congenital anomalies (Mousa et al., 2019). Fewer studies have focused directly on the impact of maternal pregnancy and lactational nutrition on fetal brain development and offspring behavior (Prado and Dewey, 2014; Li et al., 2019). There remains a knowledge gap regarding the impact of maternal micro- and macronutrient intake specifically on fetal brain development and offspring behavior.

We sought to address this knowledge gap by evaluating the impact of maternal dietary composition in pregnancy and lactation on fetal brain development and offspring behavior, in the absence of maternal pre-pregnancy obesity or maternal over- or under-nutrition, using two standard commercially available chow diets that differed significantly with respect to their micronutrient content (including vitamins and minerals), and differed in macronutrient content only with respect to carbohydrate. Our objective was to evaluate the impact of maternal pregnancy diet on fetal brain gene expression, neonatal behavior, neonatal growth trajectory, and adult offspring behavior. If standard maternal chow diets themselves have an impact on fetal brain development, the choice of chow diet may be an important variable to consider for neuroscience researchers investigating the impact of maternal exposures on the developing brain.

## Materials and Methods

### Mouse Strain, Breeding, Pregnancy and Lactation Diets

This study was part of a larger research program examining the impact of maternal nutrient supplementation in pregnancy on fetal and offspring brain development in mouse models of Down syndrome compared to wild-type (Guedj et al., 2018). Female C57Bl/6J mice (Jackson Laboratory, Bar Harbor, ME, United States) were crossed with Ts1Cje males [B6 T(12:16)1Cje/CjeDnJ], to generate pregnancies in which approximately half of the fetuses were affected with Down syndrome and half were wild-type. Only outcomes from wild-type fetuses and offspring (exposed to the intrauterine environment of a wild-type/C57Bl/6J dam) were examined. Breeder pairs received either a standard commercially available pelleted chow (Teklad 2918), or a standard commercially available purified powdered chow diet (Bioserv F3197). The content of each diet is depicted in Table 1.

**TABLE 1:** Maternal diet composition.

**Class**	**Component**	**Powder chow Bioserv F3197**	**Regular chow Teklad 2918**
**Isoflavone**	Daidzein, genistein	Not present	150–250 mg/kg
**macronutrients**	Crude protein	18.1%	18.6%
	Fat	7.1%	6.2%
	Carbohydrate	59.3%	44.2%
	Crude fiber	4.8%	3.5%
	Nutral detergent fiber	Not present	14.7%
	Ash	2.2%	5.3%
**Caloric profile**	Protein	0.72 Kcal/g	0.74 Kcal/g
	Fat	0.64 Kcal/g	0.56 Kcal/g
	Carbohydrates	2.37 Kcal/g	1.20 Kcal/g
	Total	3.74 Kcal/g	3.1 Kcal/g
**Micronutrients-Minerals**	Calcium	5.1 g/kg	10 g/kg
	Phosphorus	2.8 g/kg	7 g/kg
	Sodium	1.03 g/kg	2 g/kg
	Potassium	3.6 g/kg	6 g/kg
	Chloride	1.6 g/kg	4 g/kg
	Magnesium	0.51 g/kg	2 g/kg
	Zinc	37.7 mg/kg	70 mg/kg
	Manganese	10.5 mg/kg	100 mg/kg
	Copper	6.0 mg/kg	15 mg/kg
	Iodine	0.21 mg/kg	15 mg/kg
	Iron	37.2 mg/kg	200 mg/kg
	Selenium	0.17 mg/kg	0.23 mg/kg
	Chromium	1 mg/Kg	Not present
	Fluoride	1 mg/kg	Not present
	Sulfur	301 mg/kg	Not present
**Micronutrients-Vitamins**	Vit A	4.14 IU/g	15 IU/g
	Vit D3	1 IU/g	1.5 IU/g
	Vit E	0.083 IU/g	110 IU/g
	Vit K3	Not present	50 mg/kg
	Vit B1	6 mg/kg	17 mg/kg
	Vit B2	6 mg/kg	15 mg/kg
	Niacin	30 mg/kg	70 mg/kg
	Vit B6	5.8 mg/kg	18 mg/kg
	Pantothenic acid	14.7 mg/kg	33 mg/kg
	Vit B12	0.025 mg/kg	0.08 mg/kg
	Biotin	0.2 mg/kg	0.4 mg/kg
	Folate	2 mg/kg	4 mg/kg
	Choline	1028 mg/kg	1200 mg/kg
	Vit K1	0.88 mg/kg	Not present
**Amino acids**	Alanine	4.6 g/kg	11 g/kg
	Arginine	6.4 g/kg	10 g/kg
	Aspartic acid	11.2 g/kg	14 g/kg
	Cystine	3.5 g/kg	3 g/kg
	Glutamic acid	35.6 g/kg	34 g/kg
	Glycine	4.3 g/kg	8 g/kg
	Histidine	4.8 g/kg	4 g/kg
	Isoleucine	9.6 g/kg	8 g/kg
	Leucine	14.6 g/kg	18 g/kg
	Lysine	13.0 g/kg	9 g/kg
	Methionine	4.5 g/kg	4 g/kg
	Phenylalanine	7.8 g/kg	10 g/kg
	Proline	18.0 g/kg	16 g/kg
	Serine	10.0 g/kg	11 g/kg
	Threonine	7.7 g/kg	7 g/kg
	Tryptophan	2 g/kg	2 g/kg
	Tyrosine	10 g/kg	6 g/kg
	Valine	11.4 g/kg	9 g/kg
**Fatty acids**	C16: o Palmetic	Not present	7 g/kg%
	C18: o Stearic	Not present	2 g/kg
	C18: 1ω9 Oleic	Not present	12 g/kg
	C18:2ω6 (n-6 LC-PUFA or linoleic acid, LA)	35.7 g/kg	31 g/kg
	C18:3ω3 (n-3 LC-PUFA α-linolenic acid or ALA)	4.8 g/kg	3 g/kg
	Total saturated	11 g/kg	9 g/kg
	Total monounsaturated	15.9 g/kg	13 g/kg
	Total polyunsaturated	40.4 g/kg	34 g/kg

The rationale was to determine the effects of different chow diets on wild-type fetal brain development and offspring behavior, in order to select an optimal control diet for a subsequent set of experiments focused on the impact of isoflavone supplementation during pregnancy on fetal brain development and offspring behavior in Down syndrome (Guedj et al., 2018). The powdered chow was specifically selected for interrogation given the absence of added isoflavones. The absence of isoflavone supplementation at baseline in the powdered chow diet was of interest because this study was a precursor to an intervention study investigating the benefits of maternal dietary supplementation with apigenin, an isoflavone, on brain development in offspring with Down syndrome (Guedj et al., 2018). It was therefore important to examine the effects of a diet that could be supplemented with apigenin and did not already contain other isoflavones that could confound the evaluation of apigenin’s effect.

All fetuses and offspring reported here are wild-type fetuses exposed to the intrauterine environment of wild-type/C57Bl/6J dams that consumed one of two diets during pregnancy without additional isoflavone supplementation. A subsequent study compared brain development in mouse models of Down syndrome versus their wild-type littermates with and without isoflavone (apigenin) supplementation using only powdered chow (Guedj et al., 2018).

Dams were started on the study diet at the time of initial breeding with a sire, in order to isolate the impact of maternal dietary intake in pregnancy and lactation on fetal brain development and offspring behavior. The dams continued on the diet throughout pregnancy and lactation. Offspring were weaned to the same diet they were exposed to during pregnancy and lactation. With respect to the breeding strategy, females were bred with males overnight. Vaginal plugs were checked by 9 am the next day, with the presence of a vaginal plug defined as embryonic day 0.5 (E0.5). To exclude the possibility of daytime mating, males were separated from females during the day. Male sires were consistently fed the same single diet as per their initial breeding pair. Females were weighed at embryonic days 0 (day of mating), 10, and 15.5, just prior to euthanasia. Greater than or equal to 10% weight gain at E10 was used to confirm pregnancy (Johnson et al., 2010). Animals were housed in cages with standard bedding and nestlets. Animals were given *ad libitum* access to chow and water. The colony was maintained on a standard 12:12 light-dark cycle, with lights on at 07:00. All experiments were approved by the Tufts Medical Center Institutional Animal Care and Use Committee (IACUC, protocol #B2013-20).

### Maternal Diet Composition Differences

**FIGURE 1:**
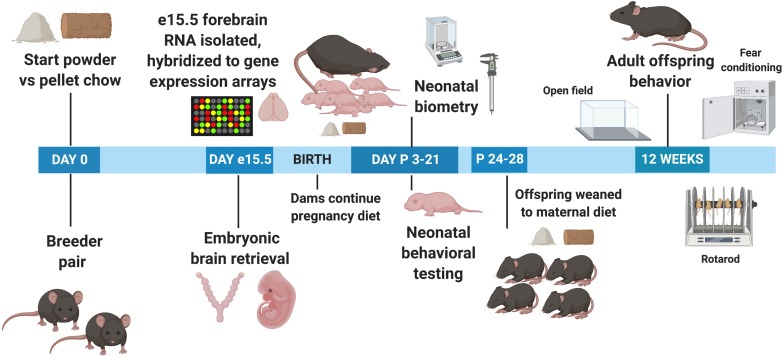
Experimental timeline. e, Embryonic day; P, Postnatal day.

Table 1 demonstrates the components of the two different chow diets. They differ significantly in several respects. The powdered chow was relatively enriched for carbohydrates compared to the pelleted diet. The powdered chow also contained significantly higher concentrations of n-3 long-chain polyunsaturated fatty acids (LC-PUFAs, linolenic acid) compared to pelleted chow, with a more favorable n-6/n-3 LC-PUFA ratio (7.4 for powdered versus 10.3 for pelleted chow, with an n-6/n-3 ratio of <10 recommended for infant and adult nutrition) (Gerster, 1998; Abedi and Sahari, 2014). The remainder of differences between the two diets favored enrichment of the pelleted diet over the powdered. Compared to the powdered chow, the pelleted chow was enriched for: (1) The isoflavones daidzein and genistein (phytoestrogens known to cross the blood-brain barrier and exert antioxidant effects) (Bang et al., 2004; Zeng et al., 2004); (2) the macroelements calcium, phosphorus, sodium, potassium, chloride, and magnesium (all two- to approximately four-fold higher in pelleted chow); (3) the amino acids alanine, arginine and glycine (all approximately two-fold higher in the pelleted chow); (4) micronutrients zinc, manganese, copper, iodine, iron, and selenium (all higher in pelleted chow, ranging from approximately 2-fold to as high as 70-fold) and vitamin content, with the pelleted chow containing universally higher vitamin concentrations than the powdered chow. With respect to micronutrients, manganese, iodine, and iron have the greatest fold differences between the two diets (9. 5-, 71-, and 5.4-fold higher in pelleted chow, respectively). With respect to vitamins, the difference between the two diets was most notable for Vitamin E (more than 1,000-fold higher in the pelleted chow). Vitamins A, B1, B2, Niacin, B6, pantothenic acid, B12, Biotin, and folate were all two- to four-fold higher in the pelleted chow. In summary, the powdered chow was relatively enriched for carbohydrate content and favorable LC-PUFAs compared to pelleted chow, but was relatively deficient in macroelements, specific amino acids, and micronutrients (including all vitamins and most minerals), compared to the pelleted chow. The experimental paradigm is depicted in Figure 1.

### Tissue Collection

On embryonic day 15.5 (E15.5), pregnant dams were euthanized with isoflurane followed by decapitation. Embryos were rapidly dissected from the uterine horns and placed in ice-cold 1X phosphate-buffered saline (PBS) containing RNA preservative (RNALater, Qiagen). Theiler staging was performed to confirm the gestational age of E15.5 (Theiler, 1989, accessed April, 2018). On a cold platform, embryonic brains were rapidly removed from skulls, and forebrain was isolated and snap frozen in liquid nitrogen, prior to storage at −80°C. Tail snips were obtained for Ts1Cje and sex genotyping. Only mice that had the wild-type genotype were used for analysis here.

### Fetal Brain Gene Expression Studies

Total RNA was isolated from embryonic forebrains using the NucleoSpin II RNA/protein kits (Machery-Nagel, Duren, Germany) per the manufacturer’s instructions. The isolation included an on-column DNase digestion step to remove genomic DNA. RNA purity, integrity and quantity were assessed using the NanoDrop ND-800 (NanoDrop, Wilmington, DE, United States) and the Bioanalyzer system (Agilent 2100; Agilent Technologies Inc., Palo Alto, CA, United States). RNA was processed and hybridized to Mouse gene 1.0 ST Arrays (Affymetrix, Santa Clara, CA, United States) as previously described (Edlow et al., 2016a; Guedj et al., 2016). Five arrays per experimental group were used, with each array corresponding to one embryonic brain. One embryo per litter from five different litters per diet group was included in microarray analyses, to minimize litter effects. Five biological replicates per group has been demonstrated to be sufficient to detect global gene expression changes in microarray studies (Allison et al., 2006; Tarca et al., 2006).

Normalization was performed using the robust multichip average algorithm (RMA) and the MBNI custom CDF^1^ version #15 for the Mouse Gene 1.0 ST array. Output consisted of data for 21,225 probe sets each corresponding to unique Entrez Gene IDs. Statistical analyses were performed using R software (version 3.1.2 or later). Fetal brain gene expression data from day 15.5 embryos of dams eating pelleted chow were compared to those from day 15.5 embryos of dams eating powdered chow.

#### Statistical Analyses: Gene Expression

Student’s *t*-test was used to identify differentially-expressed genes (DEGs) between the two diet groups. *P*-values were jointly corrected for multiple testing by calculating the Benjamini–Hochberg false discovery rate (FDR) (Benjamini, 1995). Differentially expressed genes between groups were defined as those with a raw *p*-value of <0.001, an adjusted *p*-value (FDR) of <0.01, and an absolute fold-change value of >1.5. Gene expression changes were further visualized by Principal Component Analyses (PCA) using R. The pelleted chow group was selected as the referent group; upregulated genes are more highly expressed in the brains of powdered-chow exposed embryos compared to pelleted-chow exposed embryos, downregulated genes are more lowly expressed in the brains of powdered-chow exposed embryos compared to those exposed to pelleted-chow.

Functional analysis was performed on the differentially expressed genes using Ingenuity Pathway Analysis (IPA) (Qiagen, Redwood, CA, United States). The working file for the IPA analysis may be found in Supplementary File 1. Statistical significance within IPA was defined as *p* < 0.05, and activation state was predicted based on *Z*-scores ≥ 2 (activated) or ≤−2 (inhibited), in accordance with recommended thresholds (Kramer et al., 2014; Ingenuity Systems, 2019a, b [accessed April 12, 2019]). Only pathways including three or more differentially expressed genes were considered. We also performed whole transcriptome analysis of functional gene set regulation using Gene Set Enrichment Analysis (GSEA) (Subramanian et al., 2005), with a developmentally focused annotation (the Developmental Functional Annotation at Tufts or DFLAT) (Wick et al., 2014; Edlow et al., 2015). Gene sets with an FDR *q* < 0.05 were considered significantly dysregulated.

### Offspring Biometry and Neurobehavioral Analyses

#### Neonatal Biometry and Developmental Milestone Assessment

For neonatal evaluations, 62 offspring exposed to pelleted chow *in utero* and during lactation and 31 offspring exposed to the maternal powdered chow diet were evaluated, with 1–2 offspring per sex per litter evaluated to avoid litter effects (*n* = 17 pelleted chow litters, *n* = 9 powdered chow litters). There were 15 females and 16 males in the powdered chow group, and 38 females and 24 males in the pelleted chow group. Biometry was performed on neonates from both diet groups daily from postnatal day 3 (P3) through P21 (weights), or P3-P15 (length measurements, only performed until eye opening due to limited accuracy secondary to pup movement after eye opening), to establish growth trajectories. Neonatal behavior was evaluated daily from P3 to either P15 when eye opening occurred (if this would confound the behavioral test) or P21 (weaning). Behavioral assessments started on P3 to avoid maternal stress and subsequent pup neglect or cannibalism that could impact neonatal survival and behavior. The amount of time (latency) needed to complete each test was recorded and analyzed. The neurobehavioral test protocols have been described in detail in previous publications (Hill et al., 2008; Guedj et al., 2015; Goodliffe et al., 2016). All behavioral experiments were conducted in the light phase, between 08:00 and 13:00. Test apparati were thoroughly cleaned with Sani-Cloth Plus wipes or 70% ethanol spray between mice, to minimize olfactory cues from previous trials. Mice were acclimated in the testing room in their home cages for at least 1 h prior to evaluation. For neonatal behavioral testing, pups were placed with nesting material in a bowl heated to 37°C. An investigator with extensive experience in neonatal developmental milestones performed all neonatal biometry and behavioral testing (Guedj).

A modified Fox scale (Hill et al., 2008) was used to evaluate developmental milestones in wild-type offspring exposed to powdered versus pelleted chow *in utero* and during lactation. This validated battery of behavioral tests evaluates body righting mechanisms, coordination and strength (surface righting and negative geotaxis), strength and coordination (cliff aversion and forelimb grasp), sensory system maturation (auditory startle, ear twitch, and eye opening), labyrinthine reflex (air righting), and the developmental transition from rotatory locomotion behavior to straight-line walking, reflecting the rostrocaudal development of limb coordination (open field) (Fox, 1965; Hill et al., 2008; Guedj et al., 2015, 2016; Goodliffe et al., 2016). The day of achievement of a developmental milestone was defined as the day at which the pup performed the task successfully for 2 days in a row. The time to achieve a developmental milestone (latency) and the presence or absence of a reflex was evaluated by a single investigator as stated above.

#### Adult Neurobehavioral Studies

Adult behavioral testing was performed on a subset of male offspring at 3 months of age, including 16 offspring exposed to pelleted chow *in utero* and during lactation, and 14 offspring exposed to maternal powdered chow diet. The open field test was used to evaluate locomotor activity and exploratory behavior, contextual fear conditioning was used to evaluate hippocampal learning and memory, and Rotarod test was used to evaluate motor coordination. Testing paradigms have been detailed in prior publications (Aziz et al., 2018; Guedj et al., 2018).

As previously described, open field testing utilized a 60-min trial paradigm in a 40 cm × 40 cm × 40 cm opaque plastic box, with animal tracking performed by the Ethovision 10.5 system (Noldus, Leesburg, VA, United States). The fear conditioning test was performed in a sound-attenuating cubicle with exhaust fan and stainless-steel grid floor (Med Associates, Fairfax, VT, United States), with a 5-min day one training session involving two mild foot shocks (0.5 mA for 2 s) administered at 180 and 240 s. On day two (the testing session), the mice were placed into the identical conditioning chamber for 5 min with no foot shocks, and mice were monitored for freezing (fear) behavior. Reduced freezing on day 2 was evaluated as a measure of hippocampal learning/memory deficit (failure to remember receiving a shock in the same environment on the day prior). Data were analyzed using the Freeze View software (Med Associates, Fairfax, VT, United States). Motor coordination was investigated using the rotarod test (Med Associates, Fairfax, VT, United States) using a 16 RPM, 24 RPM, and 32 RPM fixed speed protocol with two 120 s trials at each speed, separated by a 15 min inter-trial interval, as described previously (Aziz et al., 2018; Guedj et al., 2018). The latency to fall was recorded in seconds and analyzed for each mouse.

##### Statistical analyses: behavior

The Kolmogorov–Smirnov normality test and the Fisher variance equality test were performed on behavioral and biometric data to determine appropriate subsequent statistical tests. Significant differences between groups in biometry and in latency to developmental milestone achievement were evaluated via Mann–Whitney tests for single measures, or Wilcoxon signed-rank tests for repeated measures. Significant differences between groups were defined as *p* < 0.05 and *p*-values were corrected for multiple comparisons. Two-way ANOVA (maternal diet × offspring sex) was used to evaluate for the presence of significant interactions between maternal pregnancy diet and offspring sex on achievement of neonatal milestones.

### Genotyping

Genotype and sex of embryos and offspring were determined via multiplex PCR amplification of DNA extracted from tail snips (embryos) or ear punches (neonates). Cite-F/Cite R primers targeting the neomycin cassette (present only in Ts1Cje mice) were used to determine Ts1Cje or wild-type status. Sry-F/Sry-R primers directed against the *Sry* gene (present only in males) was used to determine fetal/neonatal sex. For each reaction, Fez-F/Fez-R primers were used as endogenous controls. DNA extraction and purification methods, PCR conditions, and primer information and amplicon sizes have been described in detail in previous publications (Guedj et al., 2015, 2018; Ferres et al., 2016). Only wild-type embryos and offspring were evaluated in these experiments.

## Results

### Pelleted Versus Powdered Chow Does Not Significantly Impact Dam Weight Gain During Pregnancy Nor Embryo Size

**FIGURE 2:**
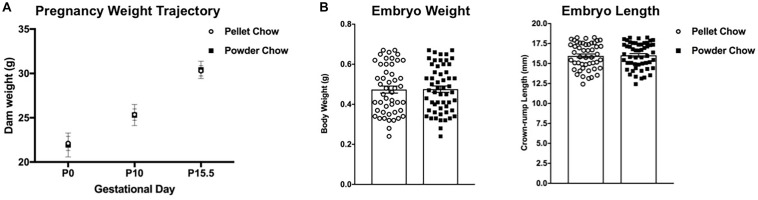
Dam weight trajectories in pregnancy, embryo weights and crown-rump lengths at embryonic day 15.5 (e15.5). There are no differences between pelleted and powdered chow for **(A)**: dam weight gain in pregnancy at pregnancy day 0 (P0, day of mating), P10 and P15 or **(B)**: embryonic (e15.5) weight (g) and crown-rump length (mm). *N* = 14 powdered chow and 14 pelleted chow litters for maternal weight gain and embryo size analyses.

There were no significant differences between maternal diet groups with respect to dam weight trajectories in pregnancy (Figure 2A). Maternal pelleted versus powdered chow diet did not have a significant impact on embryo weights or crown rump lengths at E15.5 (Figure 2B).

### Embryonic Day 15.5 Brain Gene Expression Profile Is Significantly Impacted by Maternal Pregnancy Diet Composition

**FIGURE 3:**
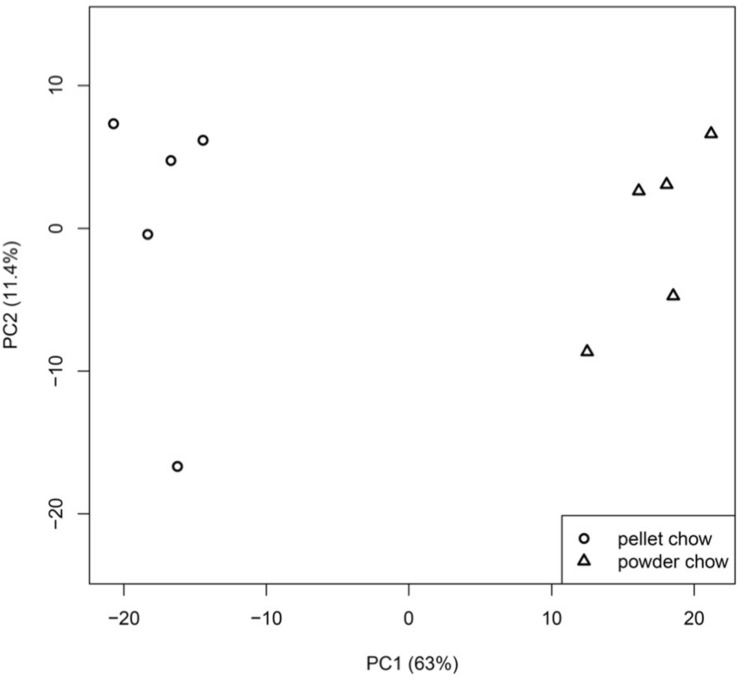
Principal component analysis (PCA) of embryonic day 15.5 brain gene expression profiles. Fetal brain gene expression clusters strongly by maternal pregnancy diet, with principal component or PC 1 (maternal pregnancy diet), accounting for 63% of the variation in fetal brain gene expression.

There were 1647 differentially-expressed genes (DEGs) in the embryonic brain exposed to maternal powdered chow diet compared to maternal pelleted chow diet. Principal component analysis demonstrated strong clustering of fetal brain gene expression by maternal pregnancy diet, with PC1 (indicating maternal pregnancy diet) accounting for 63% of the variation in fetal brain gene expression (Figure 3). Supplementary File 2 contains the list of significant DEGs including the Entrez Gene ID, gene name, chromosome location, fold change value, raw *p*- and BH-*p* values for expression levels between powdered chow-exposed embryonic brain compared to pelleted chow-exposed embryonic brain. Two-way ANOVA model (maternal diet × fetal sex) found no significant interaction effects between maternal diet and fetal sex on fetal brain gene expression, and no overlap between the genes affected by diet and those affected by sex.

#### Pathways Analyses

##### Ingenuity pathways analysis

Pathway analyses performed in IPA suggested significant potential biological impact of the dysregulated brain gene expression due to different maternal diet composition. The canonical pathway Mitotic Roles of Polo-Like Kinase was significantly upregulated in the brains of powdered-chow exposed embryos (*Z*-score 2.4, key in cell cycle regulation). Other canonical pathways that were significantly affected with an adjusted *p*-value of <0.05 but for which a definite up- or down-regulation could not be determined based on the pattern of gene expression included: (1) Protein Ubiquitination Pathway, (2) Oxidative Phosphorylation Pathway, (3) Ataxia Telangiectasia Mutated Protein (ATM) Pathway (a canonical pathway key in regulation of the cell cycle and response to cellular stress and injury), and (4) Sirtuin Signaling Pathway (canonical pathway key in regulating metabolism and energy homeostasis by controlling lipid and glucose metabolism, ketone body synthesis, urea cycle and insulin secretion). Significantly dysregulated Canonical Pathways and their constituent differentially expressed genes in the embryonic brain exposed to powdered versus pelleted chow *in utero* are depicted in Table 2.

**TABLE 2:** Significantly dysregulated canonical pathways in powdered chow-exposed fetal brain.

**Pathway and constituent genes**	**Gene name**	**Entrez gene ID (mouse)**	**Expr log ratio**	**Expr false discovery rate (q-value)**	**Location**	**Type(s)**
**Mitotic Polo-Like Kinase Pathway *p* = 7.01 × 10^−7 23 of 61 molecules in pathway dysregulated, Z-score 2.50 (upregulated)**						
ANAPC4	Anaphase promoting complex subunit 4	52206	0.603	1.60E–04	Nucleus	Enzyme
ANAPC5	Anaphase promoting complex subunit 5	59008	0.817	3.53E–06	Nucleus	Other
ANAPC13	Anaphase promoting complex subunit 13	69010	1.682	7.13E–06	Nucleus	Other
CDC7	Cell division cycle 7	12545	0.929	4.89E–05	Nucleus	Kinase
CDC16	Cell division cycle 16	69957	0.739	6.44E–05	Nucleus	Other
CDC26	Cell division cycle 26	66440	0.784	6.28E–05	Nucleus	Other
CDC25C	Cell division cycle 25C	12532	0.916	9.78E–04	Nucleus	Phosphatase
FBXO5	F-box protein 5	67141	0.911	1.06E–04	Nucleus	Enzyme
KIF11	Kinesin family member 11	16551	0.953	1.21E–03	Nucleus	Other
KIF23	Kinesin family member 23	71819	0.641	1.94E–03	Cytoplasm	Other
PLK2	Polo like kinase 2	20620	0.751	2.12E–04	Nucleus	Kinase
PLK3	Polo like kinase 3	12795	–0.649	1.33E–03	Nucleus	Kinase
PPP2R2A	Protein phosphatase 2 regulatory subunit B alpha	71978	0.64	4.54E–05	Cytoplasm	Phosphatase
PPP2R2C	Protein phosphatase 2 regulatory subunit B gamma	269643	–0.606	7.46E–05	Nucleus	Phosphatase
PPP2R3A	Protein phosphatase 2 regulatory subunit B alpha	235542	0.589	2.94E–05	Nucleus	Phosphatase
PPP2R5B	Protein phosphatase 2 regulatory subunit B beta	225849	–0.63	2.40E–05	Cytoplasm	Phosphatase
PPP2R5E	Protein phosphatase 2 regulatory subunit B epsilon	26932	0.684	1.05E–04	Cytoplasm	Phosphatase
PRC1	Protein regulator of cytokinesis 1	233406	1.286	6.32E–04	Nucleus	Other
PTTG1	Pituitary tumor-transforming 1	30939	–0.645	3.78E–03	Nucleus	Transcription regulator
SLK	STE20 like kinase	20874	0.642	5.30E–05	Nucleus	Kinase
SMC3	Structural maintenance of chromosomes 3	13006	1.111	2.99E–05	Nucleus	Other
SMC1A	Structural maintenance of chromosomes 1A	24061	0.905	5.41E–05	Nucleus	Transporter
STAG2	Stromal antigen 2	20843	0.838	1.03E–04	Nucleus	Transcription regulator
**Protein Ubiquitination Pathway *p* = 7.9 × 10^−4, 46 of 249 molecules dysregulated, Z-score NA**						
ANAPC4	Anaphase promoting complex subunit 4	52206	0.603	1.60E–04	Nucleus	Enzyme
ANAPC5	Anaphase promoting complex subunit 5	59008	0.817	3.53E–06	Nucleus	Other
B2M	Beta-2-microglobulin	12010	–1.193	4.03E–05	Plasma Membrane	Transmembrane receptor
BAP1	BRCA1 associated protein 1	104416	–0.799	3.53E–06	Nucleus	Peptidase
BIRC2	Baculoviral IAP repeat containing 2	11797	0.764	6.38E–04	Cytoplasm	Enzyme
CUL1	Cullin 1	26965	0.768	8.98E–05	Nucleus	Enzyme
DNAJC1	DnaJ heat shock protein family (Hsp40) member C1	13418	0.877	2.00E–05	Cytoplasm	Transcription regulator
DNAJC2	DnaJ heat shock protein family (Hsp40) member C2	22791	0.925	3.10E–05	Nucleus	Transcription regulator
DNAJC3	DnaJ heat shock protein family (Hsp40) member C3	100037258	0.7	8.26E–05	Cytoplasm	Other
DNAJC7	DnaJ heat shock protein family (Hsp40) member C7	56354	0.692	3.40E–05	Cytoplasm	Other
DNAJC8	DnaJ heat shock protein family (Hsp40) member C8	68598	0.903	1.09E–03	Nucleus	Other
DNAJC9	DnaJ heat shock protein family (Hsp40) member C9	108671	0.772	2.55E–04	Nucleus	Other
DNAJC10	DnaJ heat shock protein family (Hsp40) member C10	66861	0.81	4.67E–05	Cytoplasm	Enzyme
DNAJC14	DnaJ heat shock protein family (Hsp40) member C14	74330	–0.965	1.52E–05	Cytoplasm	Other
DNAJC19	DnaJ heat shock protein family (Hsp40) member C19	100503724	0.667	1.26E–03	Cytoplasm	Other
DNAJC21	DnaJ heat shock protein family (Hsp40) member C21	78244	1.18	2.94E–05	Other	Other
DNAJC30	DnaJ heat shock protein family (Hsp40) member C30	66114	–0.81	6.44E–04	Cytoplasm	Other
ELOC	elongin C	67923	–0.674	2.48E–04	Nucleus	Transcription regulator
FBXW7	F-box and WD repeat domain containing 7	50754	0.637	2.65E–05	Nucleus	Enzyme
HSPA4L	Heat shock protein family A (Hsp70) member 4 like	18415	0.794	7.77E–05	Cytoplasm	Other
MDM2	MDM2 proto-oncogene	17246	0.628	1.08E–04	Nucleus	Transcription regulator
PSMA2	Proteasome subunit alpha 2	19166	0.789	2.18E–05	Cytoplasm	Peptidase
PSMA4	Proteasome subunit alpha 4	26441	1.429	1.36E–05	Cytoplasm	Peptidase
PSMA7	Proteasome subunit alpha 7	26444	2.048	4.96E–06	Cytoplasm	Peptidase
PSMB1	Proteasome subunit beta 1	19170	0.828	2.49E–05	Cytoplasm	Peptidase
PSMB2	Proteasome subunit beta 2	26445	–0.729	1.78E–04	Cytoplasm	Peptidase
PSMB3	Proteasome subunit beta 3	26446	–1.71	1.15E–06	Cytoplasm	Peptidase
PSMB4	Proteasome subunit beta 4	19172	–0.756	2.91E–03	Cytoplasm	Peptidase
PSMB6	Proteasome subunit beta 6	19175	1.201	5.89E–05	Nucleus	Peptidase
PSMC1	Proteasome 26S subunit, ATPase 1	19179	0.732	1.05E–04	Nucleus	Peptidase
PSMC2	Proteasome 26S subunit, ATPase 2	19181	0.945	5.53E–05	Nucleus	Peptidase
PSMC5	Proteasome 26S subunit, ATPase 5	19184	0.636	1.38E–03	Nucleus	Transcription regulator
PSMC6	Proteasome 26S subunit, ATPase 6	67089	0.646	4.40E–04	Nucleus	Peptidase
PSMD7	Proteasome 26S subunit, non-ATPase 7	17463	0.589	6.59E–04	Cytoplasm	Other
PSMD11	Proteasome 26S subunit, non-ATPase 11	69077	1.09	1.99E–05	Cytoplasm	Other
PSMD12	Proteasome 26S subunit, non-ATPase 12	66997	0.636	3.26E–04	Cytoplasm	Other
PSME1	Proteasome activator subunit 1	19186	0.89	3.19E–05	Cytoplasm	Other
PSME2	Proteasome activator subunit 2	19188	–0.639	3.67E–04	Cytoplasm	Peptidase
SUGT1	SGT1 homolog, MIS12 kinetochore complex assembly cochaperone	67955	0.662	6.36E–04	Nucleus	Other
UBE2L3	Ubiquitin conjugating enzyme E2 L3	22195	–0.726	2.08E–05	Nucleus	Enzyme
UBE2V1	ubiquitin conjugating enzyme E2 V1	66589	1.141	4.21E–04	Nucleus	Transcription regulator
UBE2V2	Ubiquitin conjugating enzyme E2 V2	70620	–0.929	7.41E–05	Cytoplasm	Enzyme
UBE2Z	Ubiquitin conjugating enzyme E2 Z	268470	–0.627	2.56E–05	Nucleus	Enzyme
UCHL5	Ubiquitin C-terminal hydrolase L5	56207	0.752	1.28E–04	Cytoplasm	Peptidase
USP25	Ubiquitin specific peptidase 25	30940	0.769	3.59E–05	Cytoplasm	Peptidase
USP47	Ubiquitin specific peptidase 47	74996	0.82	3.64E–05	Cytoplasm	Peptidase
**Oxidative Phosphorylation Pathway *p*-value 0.01, 21 of 92 molecules dysregulated, Z-score 0.22**						
ATP5F1C	ATP synthase F1 subunit gamma	11949	0.775	1.50E–03	Cytoplasm	Transporter
ATP5F1D	ATP synthase F1 subunit delta	66043	–1.576	4.29E–05	Cytoplasm	Transporter
COX11	Cytochrome c oxidase copper chaperone COX11	69802	–0.958	3.03E–05	Cytoplasm	Enzyme
COX17	Cytochrome c oxidase copper chaperone COX17	12856	1.965	2.95E–06	Cytoplasm	Enzyme
COX6A1	Cytochrome c oxidase subunit 6A1	12861	0.804	3.30E–05	Cytoplasm	Enzyme
Cox6c	Cytochrome c oxidase subunit 6C	12864	–0.6	2.13E–04	Cytoplasm	Enzyme
COX7A2L	Cytochrome c oxidase subunit 7A2 like	20463	2.175	1.32E–06	Cytoplasm	Enzyme
COX8A	Cytochrome c oxidase subunit 8A	12868	–0.819	5.82E–05	Cytoplasm	Enzyme
CYB5A	Cytochrome b5 type A	109672	–0.586	3.74E–04	Cytoplasm	Enzyme
CYC1	Cytochrome c1	66445	–1.226	1.10E–04	Cytoplasm	Enzyme
NDUFA1	NADH:ubiquinone oxidoreductase subunit A1	54405	1.593	2.91E–05	Cytoplasm	Enzyme
NDUFA4	NDUFA4, mitochondrial complex associated	17992	1.402	8.33E–06	Cytoplasm	Enzyme
NDUFA6	NADH:ubiquinone oxidoreductase subunit A6	67130	0.774	1.86E–04	Cytoplasm	Enzyme
NDUFB2	NADH:ubiquinone oxidoreductase subunit B2	68198	0.751	5.90E–04	Cytoplasm	Enzyme
NDUFB6	NADH:ubiquinone oxidoreductase subunit B6	230075	–0.899	3.84E–03	Cytoplasm	Enzyme
NDUFB9	NADH:ubiquinone oxidoreductase subunit B9	66218	1.305	3.93E–06	Cytoplasm	Enzyme
NDUFB11	NADH:ubiquinone oxidoreductase subunit B11	104130	–0.749	2.27E–04	Cytoplasm	Enzyme
NDUFS4	NADH:ubiquinone oxidoreductase subunit S4	17993	–0.801	8.94E–05	Cytoplasm	Enzyme
NDUFS6	NADH:ubiquinone oxidoreductase subunit S6	407785	0.609	1.67E–03	Cytoplasm	Enzyme
NDUFS7	NADH:ubiquinone oxidoreductase core subunit S7	75406	–1.177	1.01E–05	Cytoplasm	Enzyme
UQCRFS1	Ubiquinol-cytochrome c reductase, Rieske iron-sulfur polypeptide 1	66694	0.704	2.81E–05	Cytoplasm	Enzyme
**Ataxia Telangiectasia Mutated Protein Pathway *p*-value 0.02, 20 of 89 molecules dysregulated, *Z*-score 0.24**						
CDC25C	Cell division cycle 25C	12532	0.916	9.78E–04	Nucleus	Phosphatase
CDK2	Cyclin dependent kinase 2	12566	0.678	6.59E–04	Nucleus	Kinase
GADD45A	Growth arrest and DNA damage inducible alpha	13197	0.682	6.13E–05	Nucleus	Other
GADD45G	Growth arrest and DNA damage inducible gamma	23882	0.827	3.22E–04	Nucleus	Other
MDM2	MDM2 proto-oncogene	17246	0.628	1.08E–04	Nucleus	Transcription regulator
PPM1D	Protein phosphatase, Mg2 + Mn2 + dependent 1D	53892	–0.588	5.01E–05	Cytoplasm	Phosphatase
PPP2R2A	Protein phosphatase 2 regulatory subunit Balpha	71978	0.64	4.54E–05	Cytoplasm	Phosphatase
PPP2R2C	Protein phosphatase 2 regulatory subunit Bgamma	269643	–0.606	7.46E–05	Nucleus	Phosphatase
PPP2R3A	Protein phosphatase 2 regulatory subunit B”alpha	235542	0.589	2.94E–05	Nucleus	Phosphatase
PPP2R5B	Protein phosphatase 2 regulatory subunit B’beta	225849	–0.63	2.40E–05	Cytoplasm	Phosphatase
PPP2R5E	Protein phosphatase 2 regulatory subunit B’epsilon	26932	0.684	1.05E–04	Cytoplasm	Phosphatase
RAD50	RAD50 double strand break repair protein	19360	0.626	6.95E–04	Nucleus	Enzyme
RAD51	RAD51 recombinase	19361	0.72	9.83E–04	Nucleus	Enzyme
RNF8	Ring finger protein 8	58230	0.66	7.22E–05	Nucleus	Enzyme
SMC2	Structural maintenance of chromosomes 2	14211	0.644	6.72E–04	Nucleus	Transporter
SMC3	Structural maintenance of chromosomes 3	13006	1.111	2.99E–05	Nucleus	Other
SMC1A	Structural maintenance of chromosomes 1A	24061	0.905	5.41E–05	Nucleus	Transporter
SUV39H1	Suppressor of variegation 3-9 homolog 1	20937	–0.598	2.77E–05	Nucleus	Enzyme
TLK1	Tousled like kinase 1	228012	0.803	2.21E–04	Nucleus	Kinase
TLK2	Tousled like kinase 2	24086	0.767	1.88E–04	Cytoplasm	Kinase
**Sirtuin Signaling Pathway *p-value* 0.04, 40 of 251 molecules dysregulated, *Z* = −0.76**						
ATG13	Autophagy related 13	51897	–0.765	7.34E–05	Cytoplasm	Other
ATP5F1C	ATP synthase F1 subunit gamma	11949	0.775	1.50E–03	Cytoplasm	Transporter
ATP5F1D	ATP synthase F1 subunit delta	66043	–1.576	4.29E–05	Cytoplasm	Transporter
BPGM	Bisphosphoglycerate mutase	12183	1.425	2.29E–05	Extracellular Space	Phosphatase
CYC1	Cytochrome c1	66445	–1.226	1.10E–04	Cytoplasm	Enzyme
GABARAPL1	GABA type A receptor associated protein like 1	57436	–0.756	7.58E–05	Cytoplasm	Other
GABPA	GA binding protein transcription factor subunit alpha	14390	0.632	8.98E–04	Nucleus	Transcription regulator
GADD45A	Growth arrest and DNA damage inducible alpha	13197	0.682	6.13E–05	Nucleus	Other
GADD45G	Growth arrest and DNA damage inducible gamma	23882	0.827	3.22E–04	Nucleus	Other
H1FX	H1 histone family member X	243529	–0.828	3.19E–05	Nucleus	Other
HIST1H1C	Histone cluster 1 H1 family member c	50708	–0.686	1.03E–03	Nucleus	Other
MAPK3	Mitogen-activated protein kinase 3	26417	–0.752	1.89E–04	Cytoplasm	Kinase
MAPK15	Mitogen-activated protein kinase 15	332110	–0.699	8.98E–04	Cytoplasm	Kinase
NDUFA1	NADH:ubiquinone oxidoreductase subunit A1	54405	1.593	2.91E–05	Cytoplasm	Enzyme
NDUFA4	NDUFA4, mitochondrial complex associated	17992	1.402	8.33E–06	Cytoplasm	Enzyme
NDUFA6	NADH:ubiquinone oxidoreductase subunit A6	67130	0.774	1.86E–04	Cytoplasm	Enzyme
NDUFB2	NADH:ubiquinone oxidoreductase subunit B2	68198	0.751	5.90E–04	Cytoplasm	Enzyme
NDUFB6	NADH:ubiquinone oxidoreductase subunit B6	230075	–0.899	3.84E–03	Cytoplasm	Enzyme
NDUFB9	NADH:ubiquinone oxidoreductase subunit B9	66218	1.305	3.93E–06	Cytoplasm	Enzyme
NDUFB11	NADH:ubiquinone oxidoreductase subunit B11	104130	–0.749	2.27E–04	Cytoplasm	Enzyme
NDUFS4	NADH:ubiquinone oxidoreductase subunit S4	17993	–0.801	8.94E–05	Cytoplasm	Enzyme
NDUFS6	NADH:ubiquinone oxidoreductase subunit S6	407785	0.609	1.67E–03	Cytoplasm	Enzyme
NDUFS7	NADH:ubiquinone oxidoreductase core subunit S7	75406	–1.177	1.01E–05	Cytoplasm	Enzyme
POLR1B	RNA polymerase I subunit B	20017	–0.696	2.49E–04	Nucleus	Enzyme
POLR1D	RNA polymerase I and III subunit D	20018	0.738	1.55E–04	Nucleus	Enzyme
POLR2F	RNA polymerase II subunit F	69833	–1.462	7.63E–06	Nucleus	Enzyme
PPIF	Peptidylprolyl isomerase F	105675	–0.614	2.65E–04	Cytoplasm	Enzyme
SIRT3	Sirtuin 3	64384	–0.596	1.30E–04	Cytoplasm	Enzyme
SIRT6	Sirtuin 6	50721	–1.22	8.33E–06	Nucleus	Enzyme
SLC25A4	Solute carrier family 25 member 4	11739	0.819	3.35E–05	Cytoplasm	Transporter
SMARCA5	SWISNF related, matrix associated, actin dependent regulator of chromatin, subfamily a, member 5	93762	1.405	1.36E–04	Nucleus	Transcription regulator
SOD2	Superoxide dismutase 2	20656	–0.601	6.71E–05	Cytoplasm	Enzyme
SUV39H1	Suppressor of variegation 3-9 homolog 1	20937	–0.598	2.77E–05	Nucleus	Enzyme
TIMM44	Translocase of inner mitochondrial membrane 44	21856	0.69	2.08E–05	Cytoplasm	Transporter
TIMM17A	Translocase of inner mitochondrial membrane 17A	21854	0.604	8.33E–06	Cytoplasm	Transporter
TOMM6	Translocase of outer mitochondrial membrane 6	66119	–0.93	2.99E–05	Cytoplasm	Other
TOMM70	Translocase of outer mitochondrial membrane 70	28185	0.896	4.80E–05	Cytoplasm	Transporter
TUBA4A	Tubulin alpha 4a	22145	–0.708	2.79E–03	Cytoplasm	Other
UQCRFS1	Ubiquinol-cytochrome c reductase, Rieske iron-sulfur polypeptide 1	66694	0.704	2.81E–05	Cytoplasm	Enzyme
ZIC2	Zic family member 2	22772	–0.645	1.52E–04	Nucleus	Transcription regulator

Significant activation and inhibition of several transcriptional factors and other regulatory elements of interest was predicted by the Upstream Regulator analysis within IPA (bias-corrected *Z*-score of ≥2.0 or ≤−2.0 predict significant activation or inhibition of a specific upstream regulator as described in Section “Fetal Brain Gene Expression Studies” (Ingenuity Systems, 2019a, b [accessed April 12, 2019]). Significantly activated upstream regulators in fetal brains exposed to powdered chow *in utero* included transcriptional regulators and G-protein coupled receptors implicated in negative regulation of apoptosis, regulation of the cell cycle, synaptic plasticity, brain immune and inflammatory response, sensory nervous system development, and circadian rhythm regulation. Significantly inhibited upstream regulators included transcriptional regulators, growth factors, peptidases and cytokines implicated in cognition and learning, mitochondrial apoptosis, maintenance of vascular integrity, and vasoprotection and neuroprotection in the setting of hypoxic stimuli. Significantly activated or inhibited upstream regulators and their downstream differentially expressed genes in the embryonic brain exposed to powdered versus pelleted chow *in utero* are depicted in Table 3.

**TABLE 3:** Significantly dysregulated upstream regulators and constituent downstream genes in powdered chow-exposed fetal brain.

**Upstream regulator**	**Entrez gene name**	**Putative function**	**Molecule type**	**Predicted activation state**	**Bias-corrected Z-score**	**Target molecules in dataset**
*EP400*	E1A binding protein p400	Cell cycle regulation; chromatin organization; histone H2A acetylation; histone H4 acetylation	other	Activated	2.063	*CDCA3, CENPF, FBXO5, INCENP, MCM3, SUV39H2* (direction of regulation predicts *EP400* activation in 6/6)
*ISL1*	ISL LIM homeobox 1	Encodes a member of the LIM/homeodomain family of transcription factors. The encoded protein may play an important role in regulating insulin gene expression.	Transcription regulator	Activated	2.009	*CRABP1, NPY, NXPH4, OLIG1* (direction of expression predicts *ISL1* activation in 4/4)
*PTGER2*	Prostaglandin E receptor 2	Encodes a receptor for prostaglandin E2, a metabolite of arachidonic acid. Within the brain, PGE2 receptors are involved in the regulation of synaptic activity and plasticity, in brain maturation, and are key mediators of the brain’s response to inflammation. This gene has been implicated in negative regulation of the apoptotic process	G-protein coupled receptor	Activated	3.623	*ARFGEF1, CENPE, CENPF, CEP55, CKAP2L, ECT2, KIF11, KIF15, KIF20A, KIF2C, MELK, MKI67, NUF2, PRC1, STIL, TPX2, TTK* (direction of expression predicts *PTGER2* activation in 17/17)
*POU4F1*	POU class 4 homeobox 1	Encodes a member of the POU-IV class of neural transcription factors. Implicated in sensory nervous system development, axonogenesis, and negative regulation of central nervous system apoptosis	Transcription regulator	Activated	2.013	*CHRNA3, CRABP1, NECAB2, NPY, NXPH4, OLIG1, RTN4RL2, SSTR4* (direction of expression predicts activation of *POU4F1* in 4/8)
*CLOCK*	Clock circadian regulator	Encodes a protein that is a key regulator of circadian rhythms. Polymorphisms in this gene are associated with obesity and metabolic syndrome in certain populations.	Transcription regulator	Activated	2.248	*CADM2, CLIP1, CTGF, GCC2, GTF2E1, ITM2C, LYPD6, OPN3, OSTM1, PCGF5, PIP4K2C, QSER1, THUMPD1, TIMP4* (direction of expression predicts *CLOCK* activation in 11/14 genes)
*BDNF*	Brain derived neurotrophic factor	Encodes a member of the nerve growth factor family of proteins. Inhibition of expression is associated with cognitive deficits and neurogenerative disorders such as Alzheimer’s, Parkinson’s, and Huntington’s disease.	Growth factor	Inhibited	–2.781	*ALDH7A1, ANXA5, Cdkn1c, DNAJC21, DRD2, EGR1, HSD17B4, HSPA4L mir-10, mir-154, NCDN, NPY, PCDH8, RGS4, SCCPDH, SFR1, TMED2, Tmsb4x, VAMP2, VGF* (direction of expression predicts *BDNF* inhibition in 12/20)
*IL33*	Interleukin 33	Encodes a cytokine that binds to the IL1RL1/ST2 receptor. Encoded protein is involved in the maturation of Th2 cells and the activation of mast cells, basophils, eosinophils and natural killer cells. Gene has been implicated in microglial activation and the brain’s innate immune response.	cytokine	Inhibited	–3.102	*ACAT1, ARRB1, CCL3L3, ENO2, HACD3, NFKBIB, PRPF4B, RAI14, RAMP3, RASGRP1* (direction of expression predicts *IL33* inhibition in 8/12)
*BNIP3L*	BCL2 interacting protein 3 like	Encodes a protein that belongs to the pro-apoptotic subfamily within the Bcl-2 family of proteins. The encoded protein directly targets mitochondria and causes apoptotic changes, including loss of membrane potential and the release of cytochrome c.	Other	Inhibited	–2.94	CCND3, CENPE, CENPF, CKAP2, CST3, GADD45A, GPSM2, KIF11, NT5C3A, NUF2, PRIM1, RAD54L, TOP2A (direction of expression predicts inhibition in 12/13)
*KLF3*	Kruppel like factor 3	Transcription factor that is a key regulator of adipogenesis and B cell development. KLF3 serves as a key regulator of neuronal development, and dysregulation of regulators in the KLF famiuly has been linked to has been linked to various neurological disorders. KLFs may play a key role in brain vasoprotection and neuroprotection in response to ischemic or hypoxic stimuli.	Transcription regulator	Inhibited	–2.241	AGGF1, ANXA5, CEP63, CHCHD10, CSNK1G3, EEF1AKMT1, EPRS, HNRNPH1, HYPK, IGF2BP3 (direction of expression predicts inhibition in 25/35)
*F2*	Coagulation Factor II; Thrombin	Coagulation factor II is proteolytically cleaved to form thrombin in the first step of the coagulation cascade which ultimately results in the stemming of blood loss. F2 also plays a role in maintaining vascular integrity during development and postnatal life.	Peptidase	Inhibited	–2.821	B4GALT1, BIRC2, CTGF, DOK5, EGR1, F3, PPIF, RAC1, RHOJ, SLC2A6 (direction of expression predicts inhibition in 10/12)
*C5*	Complement 5	Encodes a component of the complement system, part of the innate immune system that plays an important role in inflammation, host homeostasis, and host defense against pathogens	Cytokine	Inhibited	–2.101	CCL3L3, EFNB2, EGR1, F3, PLK3 (direction of expression predicts inhibition in 4/5)
						

Within the Downstream Effect Analysis, 56 Molecular, Cellular, and Physiological System Development Functions met criteria for significant dysregulation in the brains of powdered-chow exposed embryos compared to pelleted (*p*-value < 0.05 and involving three or more genes in the dataset). Many pathways related to cell cycle regulation were significantly dysregulated, including pathways related to the G1/S Phase, G2 Phase, G2/M transition, Mitosis, Interphase, formation of mitotic spindle, segregation of chromosomes, centrosome duplication, and cytokinesis. The function “segregation of chromosomes” within the Cell Cycle category was predicted to be significantly increased (activation *Z*-score 2.2, *p* = 0.003). Many terms related to DNA damage response were also significantly dysregulated. Other themes that emerged from the IPA Downstream Effects pathways analyses included dysregulation of pathways related to cell death (predicted to be decreased, activation *Z*-score -3.53, *p* < 0.001) and neuronal survival; to brain innate immune response (pathways related to microglia, macrophage phagocytic activity, and natural killer cell production); to synaptic transmission and plasticity (transmission in the hippocampal region was highlighted twice); to dopaminergic neuron firing and morphology; to amino acid transport (predicted to be decreased, activation *Z*-score −2.9, *p* = 0.03); and to fatty acid transport and lipid storage. All significantly dysregulated pathways and biofunctions identified by IPA in embryonic brains exposed to powdered compared to pelleted chow *in utero* are described in Supplementary File 3.

##### Gene set enrichment analysis with a development-specific annotation (GSEA/DFLAT)

Two hundred sixty-one gene sets were significantly dysregulated (FDR *q* < 0.05) in the fetal brain exposed to powdered compared to pelleted chow *in utero* (215 sets upregulated and 46 downregulated). A complete list of significantly dysregulated gene sets may be found in Supplementary File 4. GSEA/DFLAT identified many of the same dysregulated biological processes as IPA, with cell cycle dysregulation, DNA damage response, brain immune function, amino acid transport, and neurotransmitter regulation again figuring prominently.

Cell cycle regulation was the most disrupted biofunction in powdered-chow exposed brains, with more than half of the upregulated gene sets relating to cell cycle function and regulation. Representative dysregulated gene sets related to the cell cycle include chromatin assembly and disassembly, spindle formation, mitotic spindle assembly checkpoint, centromere complex assembly, regulation of chromosome segregation (and many similar gene sets), cell cycle regulation by ubiquitin-protein ligase, regulation of the metaphase to anaphase transition, M/G1 transition, sister chromatid cohesion, G2/M transition, and many others. DNA damage response and DNA processing were the second most affected biofunctions based on the number of dysregulated gene sets (double-stranded break repair, signal transduction in response to DNA damage, DNA integrity checkpoint regulation, K63-linked polyubiquitin binding, telomere maintenance, telomere organization, recombinational repair, replication fork, double-strand break repair via homologous recombination, DNA packaging and other similar), followed by RNA processing (mRNA splicing via spliceosome/multiple spliceosome-related gene sets, RNA splicing via transesterification reactions, mRNA transport and localization, ribosomal RNA metabolism and processing, mRNA export from nucleus and other similar). Immune function (both humoral and innate) was another area in which many gene sets were upregulated, including production of molecular mediators of immune response, immunoglobulin production and diversification of immune receptors, immunoglobulin-mediated immune response, somatic recombination of immunoglobulin gene segments, B cell activation involved in immune response and other similar. Multiple gene sets related to protein synthesis and function were dysregulated (primarily downregulated) including ribosome biogenesis, translational initiation and termination, downregulation of amino acid transport/amino acid transmembrane transporter activity, glutamine amino acid metabolic process, and multiple sets related to carboxylic and organic acid transporter activity. Downregulation of gene sets related to neuronal apoptosis and cell death was also noted, as were gene sets related to neurotransmitter transport, neurotransmitter regulation, and neurotransmitter receptor activity. Regulation of cAMP biosynthesis and metabolism, and adenylate cyclase activity were also downregulated in the powdered-chow exposed brains, as were gene sets related to membrane lipid metabolism, endoplasmic reticulum function, and potassium and calcium ion transport.

### Maternal Dietary Composition Has a Significant Impact on Neonatal Biometry and Behavior

#### Neonatal Biometry

**FIGURE 4:**
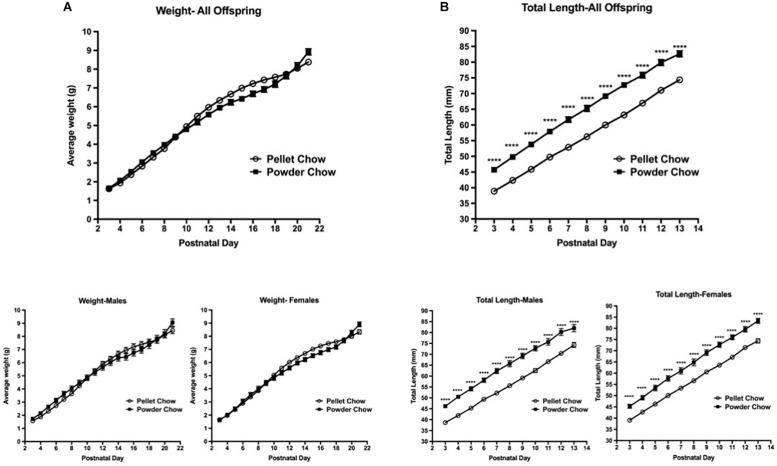
Neonatal offspring weight and length trajectory. There were no significant differences in offspring weight **(A)** between maternal diet groups, both when sexes were grouped (above) and in sex-stratified analyses (below). Powdered chow-exposed offspring were significantly longer at every postnatal day than pelleted chow-exposed counterparts **(B)**, both when sexes were grouped (above) and in sex-stratified analyses (below). *N* = 31 powder and 62 pelleted chow-exposed neonates. ^****^p < 0.0001.

Biometric analyses were performed on 31 powder-chow exposed and 62 pelleted-chow exposed neonates. These included 15 females and 16 males in the powdered chow group and 38 females and 24 males in the pelleted chow group. There was no significant difference between the two maternal diet groups with respect to offspring weight trajectory, which was true when male and female offspring were grouped and when they were examined in a sex-stratified fashion (Figure 4A). The powdered chow-exposed offspring were significantly longer at every postnatal day than pelleted chow-exposed counterparts, which was true both for grouped and sex-stratified analyses (Figure 4B).

#### Body Righting, Strength and Coordination

**FIGURE 5:**
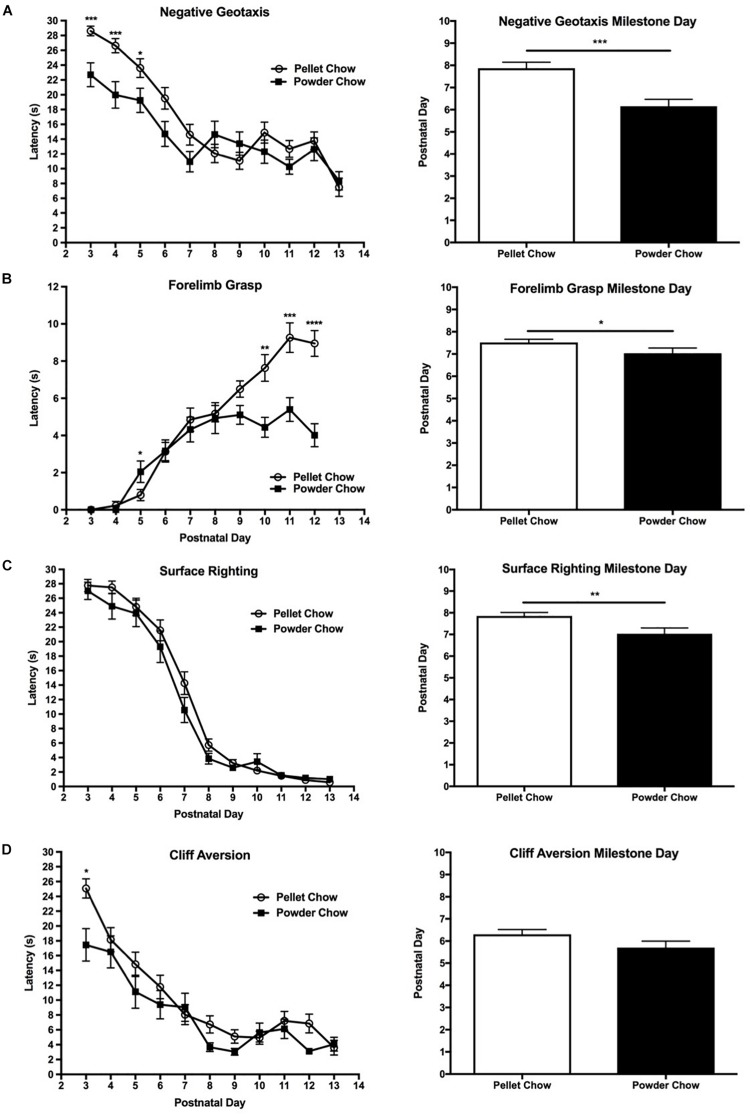
Neonatal performance on tests of coordination, strength and body righting. Powdered chow-exposed neonates achieved coordination, strength, and body-righting milestones significantly earlier than pelleted-chow exposed neonates. Graphs on the left depict neonatal latency (mean ± SEM) to complete the behavioral task by postnatal day. Graphs on the right depict differences between neonates from the two maternal diet groups in the day of developmental milestone achievement (defined as the day at which the pup performed the task successfully for 2 days in a row). **(A)** Negative geotaxis test; **(B)** Forelimb grasp test; **(C)** Surface righting test; **(D)** Cliff aversion test. *N* = 31 powder and *N* = 62 pellet-chow exposed neonates. ^∗^*p* < 0.05; ^∗∗^*p* < 0.01, ^∗∗∗^*p* < 0.001.

Powdered-chow exposed neonates achieved the body righting, strength, and coordination-related milestones significantly faster than the pelleted-chow exposed neonates. The negative geotaxis task was used to evaluate body righting mechanisms, strength, and coordination. The powdered-chow neonates had a significantly shorter daily latency on the negative geotaxis task and achieved that milestone an average of 1.7 days sooner than the pelleted-chow neonates (*p* = 0.0002, Figure 5A). Two-way ANOVA demonstrated no significant interaction terms between maternal diet and offspring sex, and no significant main effects of offspring sex on achievement of the negative geotaxis milestone [*F*_(__1_,_88__)_ = 0.65 *p* = 0.42 for interaction term, *F*_(__1_,_88__)_ = 0.14, *p* = 0.71 for offspring sex].

The forelimb grasp task was used to evaluate strength. Powdered chow-exposed offspring had significantly shorter daily latency on the forelimb grasp task, in addition to a faster overall acquisition of the forelimb grasp milestone (approximately 0.5 days earlier, *p* = 0.01, Figure 5B). Two-way ANOVA demonstrated no significant interaction terms between maternal diet and offspring sex, and no significant main effects of offspring sex on achievement of the forelimb grasp milestone [*F*_(__1_,_88__)_ = 0.87, *p* = 0.35 for interaction term, *F*_(__1_,_88__)_ = 0.04, *p* = 0.85 for offspring sex].

Surface righting task was used to evaluate body righting mechanisms, strength and coordination. The powdered-chow neonates did not have a significantly shorter latency on the surface righting task on any specific day, but achieved the surface righting milestone significantly earlier than the pelleted chow neonates (PND 7 versus 8, *p* = 0.007, Figure 5C). Two-way ANOVA demonstrated no significant interaction terms between maternal diet and offspring sex, and no significant main effects of offspring sex on achievement of the surface righting milestone [*F*_(__1_,_88__)_ = 0.65, *p* = 0.42 for interaction term, *F*_(__1_,_88__)_ = 1.31, *p* = 0.26 for offspring sex].

The cliff aversion test was used to evaluate strength and coordination. Powdered chow-exposed neonates achieved the cliff aversion milestone a mean of 0.6 days earlier than pelleted chow-exposed, but this finding did not achieve statistical significance (*p* = 0.11, Figure 5D). Two-way ANOVA demonstrated no significant interaction terms between maternal diet and offspring sex, and no significant main effects of offspring sex on achievement of the cliff aversion milestone [*F*_(__1_,_88__)_ = 0.03, *p* = 0.87 for interaction term, *F*_(__1_,_88__)_ = 1.18, *p* = 0.28 for offspring sex].

#### Transition From Rotatory/Pivoting Locomotion Behavior to Straight-Line Walking

**FIGURE 6:**
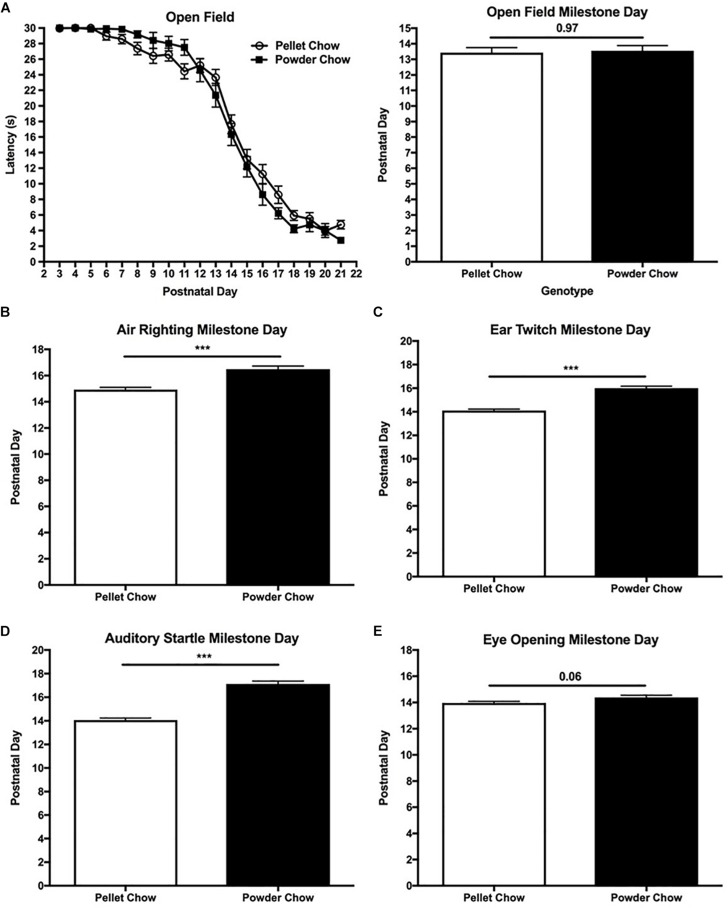
Neonatal extinction of rotatory behavior and acquisition of sensory maturation reflexes. Powdered chow-exposed neonates were significantly delayed in their achievement of sensory maturation reflexes including air righting **(B)**, ear twitch **(C)**, and auditory startle **(D)**, compared to pelleted chow-exposed neonates. Eye opening **(E)** also trended toward delay in the powdered chow-exposed neonates. There were no significant differences in neonatal extinction of rotatory behavior between maternal diet groups (evaluated by open field testing, **A**). *N* = 31 powder and *N* = 62 pellet-chow exposed neonates. ^∗∗∗^*p* < 0.001.

There were no significant differences between powdered-chow and pelleted-chow neonates with respect to daily time spent ambulating in a rotatory fashion and latency to achieve the extinction of rotatory behavior on open field testing (∼day 13 for both groups, Figure 6A). Two-way ANOVA demonstrated no significant interaction terms between maternal diet and offspring sex, and no significant main effects of offspring sex on extinction of rotatory behavior [*F*_(__1_,_88__)_ = 0.43, *p* = 0.51 for interaction term, *F*_(__1_,_88__)_ = 0.15, *p* = 0.69 for offspring sex].

#### Sensory Maturation

Unlike the body strength and coordination tasks, powdered chow-exposed neonates were significantly delayed in achievement of the sensory maturation reflexes compared to their pelleted chow counterparts. Powdered chow exposed neonates were delayed by 1.5 days in achieving the air righting reflex (evaluates the labyrinthine reflex initiated by the vestibular system, in addition to body righting and coordination) compared to pelleted chow-exposed neonates (*p* < 0.0001, Figure 6B). Two-way ANOVA demonstrated no significant interaction terms between maternal diet and offspring sex, and no significant main effects of offspring sex on achievement of the air righting milestone [*F*_(__1_,_88__)_ = 1.45, *p* = 0.23 for interaction term, *F*_(__1_,_88__)_ = 0.71, *p* = 0.40 for offspring sex]. Powdered chow offspring were 2 days delayed in achieving the ear twitch (tactile reflex, *p* < 0.0001), 3 days delayed in auditory startle (auditory reflex, *p* < 0.0001), and 0.4 days delayed in eye opening (*p* = 0.06), compared to their pelleted chow-exposed counterparts. Similar to the other neonatal milestones, two-way ANOVA demonstrated no significant interactions between maternal diet and offspring sex and no significant main effects of offspring sex on attainment of any sensory maturation reflexes [ear twitch *F*_(__1_,_88__)_ = 0.21, *p* = 0.65 for interaction term, *F*_(__1_,_88__)_ = 1.78, *p* = 0.19 for offspring sex; auditory startle *F*_(__1_,_88__)_ = 1.86, *p* = 0.18 for interaction term, *F*_(__1_,_88__)_ = 0.02, *p* = 0.88 for offspring sex; eye opening *F*_(__1_,_88__)_ = 0.35, *p* = 0.55 for interaction term, *F*_(__1_,_88__)_ = 1.48, *p* = 0.23 for offspring sex]. Figures 6C–E depicts the day of milestone achievement for powdered versus pelleted chow-exposed neonates for the aforementioned sensory maturation reflexes.

### Maternal Dietary Composition in Pregnancy and Lactation Has a Significant Impact on Adult Locomotor Activity and Hippocampal Learning

**FIGURE 7:**
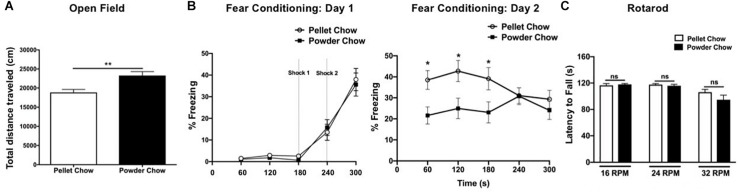
Adult offspring locomotor behavior, hippocampal learning, and motor coordination. Powdered chow-exposed adults demonstrated hyperactivity (significantly greater distance traveled in the open field test, **A**) and hippocampal learning/memory deficits (significantly reduced freezing on day 2 of contextual fear conditioning, **B**). There were no differences between groups in adult motor coordination (Rotarod testing, **C**). *N* = 14 powder and *N* = 16 pellet-chow exposed adults, except for open field where *N* = 13 powder and 16 pellet-chow exposed adults. ^∗^*p* < 0.05; ^∗∗^*p* < 0.01.

Powdered chow-exposed adult offspring traveled a significantly greater distance in the open field arena compared to pellet chow-exposed adults, consistent with hyperactivity (23,296 ± 1019 cm versus 18,853 ± 779.1 cm, *p* = 0.001, Figure 7A). Powdered chow-exposed adults demonstrated significantly reduced freezing on day 2 of fear conditioning at 60, 120, and 180 s, consistent with a hippocampal learning/memory deficit (*p* = 0.01, *p* = 0.017, and *p* = 0.039, respectively, Figure 7B). There were no significant differences between powdered chow-exposed and pellet chow-exposed adults on the rotarod test at any speed (Figure 7C).

## Discussion

In this study, we demonstrated that maternal diet in pregnancy has a significant impact on fetal brain gene expression signatures and neonatal behavior, even in the absence of overt maternal over- or undernutrition. Specifically, we found that a maternal diet relatively enriched for carbohydrates and n-3 long-chain polyunsaturated fatty acids, and relatively deficient in micronutrients, antioxidants, macroelements, and the amino acids alanine, alargine, and glycine, was associated with differential expression of over 1600 fetal brain genes, significantly increased neonatal length, earlier achievement of strength and coordination-associated milestones, and delayed neonatal sensory maturation. Importantly, these changes occurred in the absence of any differences in maternal weight gain in pregnancy or fetal weight/length changes at embryonic day 15.5, and in the absence of any neonatal weight differences, suggesting that the differences in fetal brain gene expression and neonatal behavior were not attributable to overt nutrient excess or deficiency. We also found neurobehavioral changes persisting into adult life in the offspring, with maternal powdered chow diet in pregnancy and lactation associated with hyperactivity of on open field testing, and hippocampal learning deficits on contextual fear conditioning. Both diets were commercially available “chow” diets, highlighting the critical importance of selection of appropriate control diets for those examining the impact of maternal dietary or other environmental manipulations on fetal brain development and offspring behavior.

### Impact of Dietary Micro- and Macronutrients on Brain and Organismal Development

The powdered-chow diet was relatively deficient in manganese, iodine, iron, zinc, and copper, which were 9.5-fold, 71-, 5. 4-, 2-, and 2.5-fold higher in pelleted chow, respectively. These micronutrients have all been shown to influence neurodevelopment in both human and animal model studies (Beard and Connor, 2003; Armony-Sivan et al., 2004; Hirzel et al., 2006; Corniola et al., 2008; Gao et al., 2009; Adamo and Oteiza, 2010; Gogia and Sachdev, 2012; Chung et al., 2015; Claus Henn et al., 2017). Maternal supplementation of these micronutrients has been demonstrated to improve neonatal and pregnancy outcomes, including incidence of congenital anomalies, low birth weight, low IQ or developmental delay, preterm birth, preeclampsia, and preterm premature rupture of membranes (Keats et al., 2019; Mousa et al., 2019).

It is important to note the limitations of available data on the impact of particular micronutrients on fetal and offspring brain development, particularly data from human cohorts. Key limitations include: (1) Many of the associations between maternal micronutrient deficiency and offspring neurodevelopmental outcomes are based on small observational studies that utilize maternal dietary report/recall (therefore are subject to recall bias); (2) Deficiency of a particular micronutrient is likely to co-occur with other nutrient deficiencies and potentially other conditions which may also impact fetal and offspring brain development, such as low socioeconomic status/food insecurity, poor maternal health in general, and prematurity (raising the potential for confounding). Thus, data linking any single micronutrient deficiency to adverse neurodevelopmental outcomes, particularly in observational human cohorts, should be interpreted with caution.

Maternal manganese deficiency and manganese excess have both been associated with worse performance on psychomotor and mental development indexes in human cohorts (Chung et al., 2015; Claus Henn et al., 2017). Zinc deficiency during brain development is associated with adverse neurodevelopmental outcomes in humans and in animal models, with deficits including motor delay and impairments, impaired task attention, engagement, and social behavior (Hirzel et al., 2006; Gogia and Sachdev, 2012). In rodent models, zinc deficiency is associated with cell cycle arrest, mediated in part through dysregulation of the ERK1/2, p53, and NF-kappa B pathways, and zinc deficiency during development is associated with both impaired neuronal precursor cell proliferation and induction of apoptosis (Corniola et al., 2008; Gao et al., 2009; Adamo and Oteiza, 2010). Iodine was the most discrepant micronutrient among the two experimental diets, and has been demonstrated to have a significant impact on neurodevelopment, with effects likely mediated by thyroid hormone deficiency (Skeaff, 2011). Iodine is required for neuronal growth, synapse formation, and myelination during brain development (Prado and Dewey, 2014). Iron was also significantly different between the two diets (relatively deficient in powdered chow), and has been shown to be critical to early life brain development. Iron deficiency has been associated with deficits in learning and memory in children, as well as alterations in neuron energy metabolism, dopamine signaling, and dendrite complexity in animal models (Beard and Connor, 2003; Georgieff, 2007; Prado and Dewey, 2014; Bastian et al., 2016). Particularly relevant to the deficits observed on neonatal behavioral testing in the powdered chow-exposed neonates, iron deficiency has been associated with abnormal neonatal reflexes and impaired auditory processing and auditory cortex development in human cohorts (Armony-Sivan et al., 2004; Siddappa et al., 2004).

The powdered chow was also relatively deficient in all vitamins, with vitamins A, B1, B2, Niacin, B6, pantothenic acid, B12, biotin, and folate all two- to four-fold higher in pelleted chow and Vitamin E 1000-fold higher in pelleted chow. Vitamins A, E, B6, B12 and folic acid have all been demonstrated to impact brain development, with deficiency during key developmental windows associated in human cohorts with adverse neurodevelopmental outcomes including cognitive and motor deficits, as well as autism spectrum disorder (Dias et al., 2013; Wachs et al., 2014; Altamimi, 2018; Mousa et al., 2019). Vitamin E, which was 1000-fold lower in the powdered chow diet, is known to serve as an antioxidant, with fetal neuroprotective effects demonstrated primarily inrat and hamster models in the setting of maternal toxic exposures and/or oxidative stress (Erdemli et al., 2016; Sampayo-Reyes et al., 2017; Sakamoto et al., 2018; Zhang Y. et al., 2018). Supraphysiologic/supra-nutritional maternal vitamin E consumption has been demonstrated in rodent models to induce enduring changes in hippocampal synaptic plasticity of offspring, with increased synaptic density/reduced synaptic pruning observed in the adult offspring hippocampus, and associated cognitive/learning deficits (Betti et al., 2011; Salucci et al., 2014). Thus, both Vitamin E deficiency and excess appear to have deleterious impact on the developing fetal brain.

In addition to being deficient in the antioxidants Vitamin A and E, the powdered chow lacked the isoflavones daidzein and genistein. Studies in rodent models have demonstrated an important effect of dietary soy-derived phytoestrogens on learning and memory, and in mediating anti-inflammatory and neuroprotective effects on the brain (Wang et al., 2013). Both genistein and daidzein have been demonstrated to cross the blood-brain barrier, and exert antioxidant and neuroprotective effects by several mechanisms including mediation of programed cell death and reduction of RAGE-related NF-kB activation and neurovascular production of pro-inflammatory cytokines (Zeng et al., 2004; Xi et al., 2013; Zhang Q. et al., 2018). Both isoflavones have also been demonstrated to improve cognitive function. One putative mechanism for this is upregulation of brain-derived neurotrophic factors (Pan et al., 1999; Lund et al., 2001). Daidzein has been demonstrated in rodent models to exert neuroprotective and cell-proliferative effects in the context of stroke, and in the setting of high-fat diet-induced apoptosis and gliosis (Subedi et al., 2017).

While the powdered chow was deficient for micronutrients and antioxidants, it was relatively enriched for omega-3 long-chain polyunsaturated fatty acids (in particular the n-3 LC-PUFA linolenic acid), which have been demonstrated to influence neurodevelopment. The powdered chow contained 1.6-fold higher n-3 LC-PUFAs than pelleted chow, and had a more favorable n-6/n-3 long-chain PUFA ratio than the pelleted chow (Abedi and Sahari, 2014). LC-PUFAs are fatty acids with at least 18–20 carbons, whose omega-6 (n-6) or omega-3 (n-3) designations depend on the position of the first double bond relative to the methyl end group of the fatty acid (Venegas-Caleron et al., 2010). LC-PUFAs are important in neurotransmitter synthesis and release, immune system regulation, the clotting cascade, phospholipid membrane structure in the brain and retina, and cholesterol metabolism (Li and Hu, 2009; Abedi and Sahari, 2014). N-3 LC-PUFAs are highly concentrated in the mammalian retina and brain, and play a key role in normal visual and brain function due to their involvement in neurotransmitter biosynthesis, signal transduction, and monoamine neurotransmitter receptor binding and activity (Li and Hu, 2009). The powdered chow also had a higher content of carbohydrates when compared with the pelleted chow. There is a dearth of information in the literature about the impact of dietary carbohydrates on neurodevelopment, although overall maternal dietary quality, one measure of which includes total caloric consumption, has been demonstrated to be associated with neurodevelopmental outcomes (Malin et al., 2018). The slight increase in calories in the powdered chow (3.7 kcal/g versus 3.1 kcal/g in the pelleted chow), which is primarily attributable to the increased carbohydrate content, could impact overall organismal development, including of the developing brain.

### Pathways Analysis of Fetal Brain Transcriptome Data in Context

Key themes in the functional analyses of differentially expressed fetal brain genes included dysregulation of the cell cycle, of DNA damage response, and of apoptosis. Significant upregulation of the canonical pathway Mitotic Roles of Polo-Like Kinase (key in cell cycle regulation), and significant dysregulation of multiple downstream pathways implicated in cell cycle regulation, was noted in the powdered chow-exposed fetal brain. The polo-like kinases are a highly conserved family of proteins identified in yeast, *Xenopus*, *C. elegans* and mammals, playing a key regulatory role for entry into and exit from mitosis, centrosome separation and maturation, and promoting the onset of cytokinesis, among other functions (Glover et al., 1998; Donaldson et al., 2001). Polo-like kinases have been implicated in neurogenesis and synaptic plasticity (Kauselmann et al., 1999; Sakai et al., 2012; Genin et al., 2014), suggesting that activation of polo-like kinases might reflect increased demand for new neural progenitor cells or new neuronal connections. Whether the significant upregulation of this canonical pathway means that there is increased flux through the cell cycle in the brains of powdered chow-exposed embryos, and if there is increased flux, whether this reflects increased neurogenesis, increased cell death, or both, is beyond the scope of these experiments to determine. These results point to the need for additional experiments investigating the impact of micronutrient deficiency on cellular proliferation and death in the developing brain, specifically as these processes relate to increased flux through the cell cycle mediated by polo-like kinases.

DNA damage response was another key dysregulated biological process in the functional analyses, and might be related to the significant dysregulation of cell-cycle related biological functions and canonical pathways. There are numerous examples of micronutrient deficiencies associated with DNA damage (Ames, 1999). In particular, iron is an essential cofactor for proteins which regulate DNA replication, repair, and cell cycle progression (Zhang, 2014; Jung et al., 2019). One such protein for which iron is an essential cofactor is the enzyme GADD45, which was noted to be significantly dysregulated in the powdered chow-exposed embryonic brains, and is involved in cell cycle arrest after DNA damage (Gao et al., 1999; Steegmann-Olmedillas, 2011). Dietary zinc supplementation has also been linked with reduced DNA strand breaks in human subjects; proteins involved in DNA repair, antioxidant, and immune functions were restored after dietary zinc was increased (Song et al., 2009; Zyba et al., 2017).

Negative regulation of apoptosis in the powdered chow-exposed fetal brain was another key theme in the functional analyses, including activation of both upstream regulators that downregulate apoptosis, and downstream pathways that negatively regulate apoptosis. This finding is consistent with gene expression patterns suggesting reduced apoptosis in the human fetal brain exposed to maternal obesity, which may itself be a state of relative micronutrient deficiency (Edlow et al., 2014). Multiple micronutrients that are deficient in the powdered chow diet have been linked to increased apoptosis in animal models, including iodine, manganese, zinc, and copper (Beard and Connor, 2003; Armony-Sivan et al., 2004; Vanlandingham et al., 2005; Georgieff, 2007; Tamm et al., 2008; Gao et al., 2009; Cui et al., 2018). Zinc deficiency, in particular, is strongly associated with apoptosis and abnormal neural progenitor regulation in the developing brain (Corniola et al., 2008; Gao et al., 2009; Adamo and Oteiza, 2010). Thus, downregulation of apoptosis-related pathways in the powdered chow-exposed fetal brain could be compensatory. Determining whether apoptosis is up- or down-regulated in the powdered chow-exposed/relatively micronutrient-deficient brain, and whether the downregulation of apoptosis seen on pathways analysis is compensatory or causative are important directions for future investigation.

Other key themes in both the IPA and GSEA/DFLAT analyses included decreased amino acid transport in the powdered chow-exposed fetal brain; dysregulated synaptic transmission and plasticity; brain innate immune response (including pathways related to microglia/macrophage phagocytic activity, as well as killer cell activity); and dopaminergic signaling. The changes in amino acid transport pathways could reflect the powdered chow’s relative deficiency of multiple amino acids, especially glycine, alanine and arginine. Several components deficient in the powdered chow diet have been demonstrated to play an important role in synaptic plasticity, in particular zinc and iron (Beard and Connor, 2003; Jorgenson et al., 2005; Georgieff, 2007). LC-PUFAs, which are relatively enriched in the powdered chow diet, have also been demonstrated to play an important role in synaptogenesis, learning and memory, and synaptic plasticity (Georgieff and Innis, 2005; Georgieff, 2007; Crupi et al., 2013). Deficiency of iron, zinc, and copper, and reduced antioxidant capacity conferred by relative deficiency of antioxidants (including the specific isoflavones daidzein and genistein), have been associated with altered microglial function and brain innate immune response (Cunningham-Rundles et al., 2009; Ibrahim et al., 2010; Chinta et al., 2013). Iron and copper deficiency (both relatively deficient in the powdered-chow exposed diet) have been demonstrated to impact dopamine synthesis, metabolism and release in the striatum (Prohaska and Brokate, 2001; Beard and Connor, 2003; Georgieff, 2007).

### Contextualizing Neonatal Biometric Parameters and Behavioral Testing

The multiple differences between the two diets preclude attribution of neonatal growth changes or performance on any particular behavioral test to relative excess or deficiency of a particular dietary component. One possible interpretation of the neonatal length and behavioral differences between diet groups, however, is that the increased length and improved attainment of strength-and coordination-related tasks in the powdered chow-exposed pups may be attributable to aspects of the diet that were relatively enriched in powdered chow, while the delay in sensory milestone achievement might be attributable to relative deficiencies in the powdered chow diet. Thus, the relative enrichment of powdered chow for the n-3 LC-PUFA linolenic acid and carbohydrates could be a driver for increased length and improved strength and coordination in the pups, while the relative deficiency in macroelements, micronutrients and antioxidants could be a driver of the delays in sensory maturation noted.

Although our study design does not permit the explicit testing of these hypotheses, the existing literature suggests there is biologic plausibility for both omega-3 LC-PUFAs and carbohydrate enrichment increasing body length, and for omega-3 LC-PUFA enrichment improving strength and coordination. Prior studies have found an association between omega-3:omega-6 maternal dietary ratios and neonatal length in mice (Santillan et al., 2010). Maternal diet correlates with neonatal length in humans, as well (Rodriguez-Bernal et al., 2010; Hjertholm et al., 2018). Associations have also been noted between maternal dietary carbohydrate content and offspring body size, although weight and/or fat mass is more frequently reported to be significantly increased in the setting of increased carbohydrate consumption, compared to length (Renault et al., 2015; Crume et al., 2016).

LC-PUFAs are highly transferred between mother and neonate in breast milk and play a key role in synaptogenesis, synaptic plasticity, and myelination (Georgieff, 2007; Crupi et al., 2013). The majority of animal studies have demonstrated omega-3 fatty acid supplementation is associated with improvement in cognitive tasks, visual acuity, neurogenesis, and other brain-development-related endpoints (Lauritzen et al., 2016). Studies evaluating the benefit of maternal omega-3 fatty acid supplementation on cognition in humans, however, have been mixed. Inconsistent with our results demonstrating sensory maturation deficits in powdered chow-exposed offspring, a study in Turkish infants demonstrated that DHA-enriched formula was associated with more rapid acquisition of brainstem auditory-evoked potentials (Unay et al., 2004). Consistent with our results demonstrating improved coordination in powdered-chow exposed offspring, a small randomized trial found that omega-3 fatty acid supplementation was associated with improved hand-eye coordination in 2.5-year-old children (Dunstan et al., 2008). However, two comprehensive reviews, including one of nine randomized controlled studies, found no consistent sustained benefit of omega-3 supplementation for infant cognition or visual development (Lo et al., 2012; Prado and Dewey, 2014). Overall, studies evaluating the benefit of maternal omega-3 fatty acid supplementation on offspring developmental outcomes have demonstrated the most benefit in preterm infants (SanGiovanni et al., 2000; O’Connor et al., 2001; Makrides et al., 2010).

With respect to strength, omega-3 LC-PUFAs have been demonstrated to improve muscle contractility in rats (Patten et al., 2002), and in humans when omega-3 fatty acid supplementation was combined with an exercise and resistance-training regimen (Rodacki et al., 2012). This may be mediated by enhanced sensitivity of the muscle to acetylcholine, a neurotransmitter that stimulates muscle contraction (Jeromson et al., 2015). Finally, it is possible that the increased body length itself had a favorable impact on strength and coordination-related neonatal behavioral tasks; this association has not been previously reported, and may be an interesting direction for future study.

There is also biologic plausibility for micronutrient and antioxidant deficiency impacting sensory maturation. In a guinea pig model, mild maternal iron deficiency during pregnancy and lactation was associated with abnormal auditory function in postnatal day 24 offspring (Jougleux et al., 2011). Maternal iodine deficiency, even if mild, has also been associated with disorders of auditory processing in human offspring (Azizi et al., 1995; Hynes et al., 2013), and in rodents, early postnatal iodine deficiency and hypothyroidism resulted in decreased dendritic branching in the visual and auditory cortex (Dussault and Ruel, 1987). Maternal and neonatal zinc deficiency has also been associated in multiple studies with abnormal development of brain regions that play critical roles in processing sensory information (Hagmeyer et al., 2014), and with neurosensory disorders in children (Prasad, 1996). Although maternal and perinatal zinc deficiency has more classically been associated with deficits in offspring learning and memory and social behavior (Golub et al., 1995; Hagmeyer et al., 2014), the brain areas rich in zinc-containing glutamatergic neurons (cerebral cortex and limbic structures) also play a key role in sensory processing (Hagmeyer et al., 2014). With respect to ways in which relative antioxidant deficiency may contribute to deficits in sensory maturiation, early life oxidative stress has been demonstrated to impair the development of parvalbumin-expressing fast-spiking interneurons, leading to deficits in sensory processing (Cabungcal et al., 2013).

### Contextualizing Adult Offspring Neurobehavioral Testing

With respect to the finding of hippocampal learning deficits in powdered-chow exposed adult offspring, multiple human studies have demonstrated relative micronutrient deficiency negatively impacts offspring cognition, with micronutrient supplementation of pregnant women and their infants/children associated with improved child cognition (standardized reading and math scores and information processing measures) (Prado and Dewey, 2014). However, these data are limited by concomitant increased caloric intake and/or improved protein intake during the period of supplementation for most studies, and lack of standardization of micronutrient supplementation content. While nearly all the micronutrients and antioxidants relatively deficient in the powdered-chow diet have been linked to hippocampal development and learning/memory, the strongest data in human and animal model studies are for iron and iodine deficiency in early development resulting in abnormal hippocampal development and cognitive and learning deficits that endure into adult life (Dussault and Ruel, 1987; Jorgenson et al., 2003, 2005; Lozoff et al., 2006; Beard, 2007; de Escobar et al., 2007; Walker et al., 2007; Fretham et al., 2011; Prado and Dewey, 2014).

With respect to the finding of hyperactivity/increased locomotion in powdered-chow exposed offspring, human studies evaluating the impact of vitamin and micronutrient deficiencies on offspring attention deficit hyperactivity disorder (ADHD) risk have reported mixed results (Li et al., 2019). For example, maternal multivitamin and folate intake were associated with a lower risk of ADHD diagnosis and medication use in the Danish National Birth Cohort, but not in a New Zealand birth cohort (Virk et al., 2018; D’Souza et al., 2019). Deficiencies in B vitamins and Vitamin D have also been linked to increased ADHD risk in children (Morales et al., 2015; Altun et al., 2018; Fasihpour et al., 2019; Kotsi et al., 2019), although some studies have failed to find these associations (Gustafsson et al., 2015). Inconsistent with our finding of hyperactivity in the powder-chow exposed offspring, recent human and rodent studies have reported that increased cord blood n-6/n-3 fatty acid ratio or relative maternal deficiency of n-3 fatty acids is associated with increased risk for ADHD in offspring (Fedorova and Salem, 2006; Sakayori et al., 2016; Lopez-Vicente et al., 2019), A recent systematic meta-analysis concluded that maternal omega-3 fatty acid intake has not been consistently associated with offspring ADHD risk, however (Li et al., 2019). Poor maternal dietary quality, particularly diets high in sugar, fat and processed foods (which may in some cases be a marker for micronutrient deficiency), has been more consistently linked to ADHD risk in offspring (Rijlaarsdam et al., 2017; Galera et al., 2018). Animal model studies have demonstrated more consistent associations between micronutrient deficiency during development and hyperactivity of offspring, with relative deficiency of B vitamins and iron most strongly implicated (Lalonde et al., 2008; LeBlanc et al., 2009; Fiset et al., 2015).

### Strengths, Limitations and Future Directions

As one of the first studies to directly examine the impact of maternal micronutrient and antioxidant deficiency on fetal brain development and offspring behavior in the absence of overt maternal over- or undernutrition, this study begins to address a significant knowledge gap. Pathway analysis of differentially expressed fetal brain genes provided unique insights into biological processes that could be impacted by the relative excess and deficient dietary components in the powdered chow. We hope that the gene expression changes and dysregulated pathways highlighted here can act as a starting point for future studies designed to examine the impact of specific combinations of maternal dietary components on fetal neurodevelopment and offspring behavior. The use of multiple pathways analysis tools, including a tool with annotation specific to fetal development (DFLAT), provides the most comprehensive picture of dysregulated biological processes (Edlow et al., 2015). The use of a comprehensive and validated battery of neonatal behavioral assessments performed by a single experienced investigator is also a strength. The multiple dietary components that differ between the study diets is a limitation, as it does not permit us to attribute specific gene expression changes or putative biological functional changes to particular macro- or micronutrient components, only to demonstrate that “chow” diet composition has a profound impact on the developing fetal brain and enduring consequences for offspring behavior. However, as a recent publication notes, studies that examine the impact of deficiency or enrichment for a single micro- or macronutrient have limited “real world” applicability, and to maximize translatability, studies should focus on the neurodevelopmental impact of combinations of nutrients, given that nutrients are actually ingested in combination (Malin et al., 2018). Because offspring were weaned to the same diet they were exposed to *in utero* and during lactation, we cannot determine whether the adult behavioral differences were attributable solely to the intrauterine and lactational environment, the postnatal diet exposure, or a combination of the two. Although the study was not designed to interrogate the contribution of paternal diet, a strength of the study design is that male sires were exposed to the same diet as the dams, and male sires were kept on a powdered or pelleted chow diet throughout the duration of the study, with no crossover of male sires between maternal diet groups.

The relatively small number of microarrays per maternal diet group could be viewed as a potential limitation, but five biological replicates per group is sufficient in microarray studies to detect differences in global gene expression (Allison et al., 2006; Tarca et al., 2006). While this number of microarrays is sufficient to interrogate the impact of maternal diet on fetal brain gene expression, the study was not specifically designed or powered to evaluate sex differences in fetal brain gene expression in the setting of this maternal exposure. Therefore, the lack of a significant sex-diet interaction on two-way ANOVA modeling of the differentially expressed brain genes should be interpreted with caution. One piece of evidence in favor of no actual fetal sex effect is the additional lack of a significant effect of offspring sex in the neonatal behavioral analyses. The large number of offspring per sex in the neonatal behavioral experiments (15–37/sex/maternal diet group) suggests that in the context of this particular maternal dietary exposure, fetal and offspring sex truly may not be a significant modifier. Additional studies designed and powered specifically to look at the impact of fetal sex in the setting of these maternal dietary exposures are necessary to definitively exclude fetal sex as an effect modifier. Other future directions include specific enrichment or depletion of maternal diet for only one or a small combination of micro- or macronutrients to explicitly test some of the hypotheses put forward here regarding the impact of LC-PUFA and carbohydrate enrichment on neonatal length, strength and coordination, and the impact of relative micronutrient and antioxidant deficiency on neonatal sensory maturation. In addition, specific evaluation of the mother’s milk for particular dietary components would be a useful adjunct to future studies.

## Summary

In this study, we demonstrate the important role of maternal diet composition in fetal brain development and offspring behavior, even in the absence of maternal over- or undernutrition. We show that commercially available chow diets are not interchangeable, and the content of maternal chow diet significantly impacts embryonic brain gene expression, neonatal behavior, and adult behavior in mice. These findings underscore the importance of selecting a maternal chow diet matched for macro- and micronutrients when investigating fetal and offspring brain development and behavior.

## Data Availability Statement

The datasets generated for this study can be found in the GEO, accession number GSE133525.

## Ethics Statement

The animal study was reviewed and approved by the Tufts Medical Center IACUC.

## Author Contributions

AE, FG, and DB contributed to conceiving and designing the study. AE, FG, and JP performed the statistical analysis of behavioral and microarray data. FG performed the neonatal behavioral and microarray experiments. AE performed *in silico* microarray experiments and wrote the first draft of the manuscript. DS wrote sections of the manuscript. All authors contributed to the manuscript revision, and read and approved the submitted version.

## Conflict of Interest

The authors declare that the research was conducted in the absence of any commercial or financial relationships that could be construed as a potential conflict of interest.

## Footnotes

1 http://brainarray.mbni.med.umich.edu/
