# Supplementary material for: Significant Effects of Maternal Diet During Pregnancy on the Murine Fetal Brain Transcriptome and Offspring Behavior
Source: Front Neurosci. 2019 Dec 17;13:1335. doi: 10.3389/fnins.2019.01335 (PMC6928003; doi:10.3389/fnins.2019.01335)
Supplement: Supplementary file 3 [file Table_3.DOCX]

**Supplementary File 3: Significantly Dysregulated Downstream Pathways and Biofunctions in IPA***

| **Categories** | **Diseases or Functions Annotation** | **P-value** | **Predicted Activation State** | **Activation Z-score** | **Molecules** |
| --- | --- | --- | --- | --- | --- |
| Cancer,Cell Death and Survival,  Organismal Injury and Abnormalities,  Tumor Morphology | Cell death | 9.75E-05 | Decreased | -3.53 | CRNKL1,EBNA1BP2,EIF3E,EIF3F,GPN1,KPNB1,PHF5A,PSMA4,PSMA7,PSMB1,PSMB3,PSMC1,PSMC2,PSMC5,PSMC6,PSMD11,PSMD12,RPL13A,RPL27A,RPS21,SF3B2,SF3B6,SMC1A,SUPT16H,TTK,U2af1 |
| Cardiovascular System Development and Function,Cellular Assembly and Organization,Cellular Development,Cellular Growth and Proliferation,Embryonic Development,Organ Development,Organismal Development,Skeletal and Muscular System Development and Function,Tissue Development | Formation of myofibrils | 6.86E-04 | Increased | 2.138 | AKAP13,COL4A3BP,DIAPH3,DNMT3A,FMNL2,MAP2K6,MAP2K7,TXN |
| Cellular Assembly and Organization | Binding of telomeres | 7.71E-04 |  |  | SUV39H1,SUV39H2,TERF1 |
| Lipid Metabolism,Molecular Transport,Small Molecule Biochemistry | Concentration of docosahexaenoic acid | 9.09E-04 |  | -1.982 | FABP5,FADS2,SLC27A4,SNCA |
| Cell Cycle,Cell Morphology | Formation of mitotic spindle | 9.27E-04 |  | -1.982 | FBXO5,HELLS,KIF11,KIF2C,KPNB1,MDM2 |
| Cell Cycle,Cellular Assembly and Organization,DNA Replication, Recombination, and Repair | Segregation of chromosomes | 2.67E-03 | Increased | 2.2 | ATRX,CUL1,HMMR,KIF2C,NDC80,STAG1,TPX2 |
| Neurological Disease,Psychological Disorders | Schizophrenia | 2.85E-03 |  |  | CACNG2,CCK,CLTB,CNP,CRYM,CSRP1,FNTA,GLRB,GRINA,GRM3,GSK3A,HCN3,HPRT1,LEMD2,LRRC8B,mir-154,NDUFA6,NPY,NREP,OXCT1,PCDH8,PCP4,PFDN6,PNPLA8,RGS4,RTF1,RTN2,RUSC1,S100A1,SLC7A11,SNCB,TRANK1,UBE2V2,VGF |
| Amino Acid Metabolism,Molecular Transport,Small Molecule Biochemistry | Uptake of L-triiodothyronine | 2.87E-03 |  |  | CRYM,SLC3A2,SLC7A5 |
| Cellular Assembly and Organization | Formation of vacuole | 4.59E-03 |  |  | CCK,HEPACAM,HLTF,IDS,LMNB1,MAN2C1,NDUFS4,SOD2 |
| Neurological Disease,Organismal Injury and Abnormalities | Epilepsy | 4.59E-03 |  | 0 | CHRNA4,GABRA3,LUC7L3,NCDN,NTF3,RAC1,RAC3,SSTR1 |
| Cell-mediated Immune Response | Production of natural killer cells | 6.10E-03 |  | 0.762 | EOMES,ID2,IKZF5,IL18 |
| Cellular Assembly and Organization,DNA Replication, Recombination, and Repair | Quantity of chromosomes | 6.10E-03 |  | -1 | CDK2,CENPE,GADD45A,PTTG1 |
| Cellular Development,Cellular Growth and Proliferation,Nervous System Development and Function,Tissue Development | Outgrowth of ganglion cells | 6.69E-03 |  |  | NTF3,RTN4,RTN4RL2 |
| Lipid Metabolism,Molecular Transport,Small Molecule Biochemistry | Accumulation of ceramide | 6.69E-03 |  |  | SIRT3,SMPD1,SMPD2 |
| Cell Cycle | G2/M phase | 7.52E-03 |  | 0 | ATRX,Calm1 (includes others),CDC25C,CDK2,CENPJ,CEP135,CEP290,CEP57,CEP63,CEP70,CLSPN,CNTRL,CUL1,DCTN3,GADD45A,GADD45G,HAUS3,HAUS6,HDAC3,MELK,MIIP,NOP53,PLK3,PPM1D,PPME1,PPP2R2A,RNASEH2B,SYF2,TAOK3,TERF1,TUBA4A |
| Cell Cycle | Mitosis | 7.86E-03 |  | -1.59 | AATF,ANLN,ARRB1,B4GALT1,CCK,CDC16,CDC25C,CDK11A,CDK13,CDK2,Cdkn1c,CENPC,CENPE,DBF4,DRD2,EGR1,FBXO5,GADD45A,GADD45G,GRP,HDAC3,HELLS,INCENP,INTS13,KDM5B,KDR,KIF11,KIF2C,KNTC1,KPNB1,MAP9,MAPK3,MDM2,MKI67,NDC80,PEBP1,PHIP,PTX3,RAC1,RAD50,TERF1,TMEM8B,TXN,ZWINT |
| Cell Cycle | G2 phase | 8.07E-03 |  | -0.728 | ATRX,Calm1 (includes others),CDC25C,CDK2,CENPJ,CEP135,CEP290,CEP57,CEP63,CEP70,CLSPN,CNTRL,CUL1,DCTN3,EGR1,GADD45A,GADD45G,HAUS3,HAUS6,HDAC3,MAP2K6,MELK,MIIP,MSH2,NOP53,PLK3,PPM1D,PPME1,PPP2R2A,RAD51,RNASEH2B,SYF2,TAOK3,TERF1,TUBA4A |
| Lipid Metabolism,Molecular Transport,Small Molecule Biochemistry | Transport of fatty acid | 9.43E-03 |  | -1.954 | SLC13A3,SLC6A1,SLC6A11,SLC6A13 |
| Cell Cycle | Interphase of connective tissue cells | 1.08E-02 |  | -0.096 | ATRX,CDC25C,CDC7,CDK2,DDIAS,GADD45A,ID2,MAP2K6,MAPK3,MDM2,PLK2,RAC1,RNASEH2B,TFDP1 |
| Cell-To-Cell Signaling and Interaction,Nervous System Development and Function | Neurotransmission of hippocampal CA1 region | 1.22E-02 |  | 0.555 | CDK5,CHRM2,EIF4EBP2,GRM5,Nrgn,SLC7A11 |
| Cellular Assembly and Organization,Cellular Compromise,Cellular Function and Maintenance | Destabilization of microtubules | 1.25E-02 |  |  | EGR1,KIF2C,TACC3 |
| Cancer,Organismal Injury and Abnormalities,Reproductive System Disease | Uterine tumor | 1.25E-02 |  |  | F3,PHB,TRA2B |
| Cell Cycle | Interphase of fibroblasts | 1.31E-02 |  | 0.2 | CDC25C,CDC7,CDK2,DDIAS,GADD45A,ID2,MAP2K6,MAPK3,MDM2,PLK2,RAC1,RNASEH2B,TFDP1 |
| DNA Replication, Recombination, and Repair | DNA damage response of cells | 1.53E-02 |  |  | APTX,ATAD5,BCCIP,BOD1L1,CDIP1,CHD2,CIB1,DDIAS,FANCF,FANCG,FBXO45,GADD45A,HUS1,MAPK15,MAPK3,MSH2,NEK1,NIPBL,NOP53,PAXX,PHLDA3,PLK3,RAD50,RAD51,RAD54L,RNF8,SLF1,SMC6,SPRTN,SUV39H1,TAOK3,TLK2,TOP2A,UBR5,USP47 |
| Cell Cycle | G2/M phase transition | 1.54E-02 |  | -0.555 | Calm1 (includes others),CDC25C,CDK2,CENPJ,CEP135,CEP290,CEP57,CEP63,CEP70,CNTRL,CUL1,DCTN3,GADD45A,GADD45G,HAUS3,HAUS6,MELK,MIIP,PLK3,PPM1D,PPME1,PPP2R2A,RNASEH2B,TUBA4A |
| Cell Morphology,Organismal Injury and Abnormalities | Swelling of cells | 1.68E-02 |  |  | CASP1,CNP,IL18,KCNJ10,LMNB1,MAN2C1,OSTM1,RAC1,ROCK1 |
| Neurological Disease,Organismal Injury and Abnormalities | Seizure disorder | 1.71E-02 |  | 0.478 | CHRNA4,GABRA3,KCNJ10,LUC7L3,NCDN,NR2F1,NTF3,RAC1,RAC3,SSTR1,VAMP2 |
| Cell Cycle,Cellular Movement | Cytokinesis | 1.80E-02 |  | 1.722 | ANLN,CSPP1,DRD2,ECT2,GADD45A,HMMR,INCENP,KIF20A,KIF20B,KIF23,MAP9,mir-10,PKN2,PLK3,PRC1,PRPF40A,RAB35,Spg20,TM4SF1 |
| Hematological Disease,Immunological Disease,Infectious Diseases | Infection of macrophages | 1.88E-02 |  | -1.016 | CASP1,EIF2AK2,IL18,PSIP1,RNF41,SEMA7A |
| Cell Cycle | Interphase | 1.89E-02 |  | -0.012 | ANGPT1,Anp32b,ATRX,Calm1 (includes others),CAMK2G,CCND3,CDC25C,CDC7,CDK2,Cdkn1c,CENPJ,CEP135,CEP290,CEP57,CEP63,CEP70,CLSPN,CNTRL,CUL1,CUL4A,DBF4,DCTN3,DDIAS,EGR1,EIF2AK2,FBXO5,GADD45A,GADD45G,HAUS3,HAUS6,HDAC3,ID2,KDR,MAP2K6,MAPK3,MCM3,MDM2,MELK,MIIP,MSH2,NASP,NOCT,NOP53,NPM1,ORC4,PLK2,PLK3,POLA1,POLE3,PPIF,PPM1D,PPME1,PPP1CA,PPP2R2A,PRIM1,RAC1,RAD51,RCHY1,RNASEH2B,SMAD7,SMC1A,SOD2,STX4,SYF2,TAOK3,TERF1,TFDP1,TUBA4A |
| Cell Cycle | M phase | 1.90E-02 |  | 1.428 | ANLN,CDK2,CSPP1,DRD2,ECT2,FBXO5,GADD45A,HMMR,INCENP,KIF20A,KIF20B,KIF23,MAP9,mir-10,PKN2,PLK3,PPM1D,PRC1,PRPF40A,PTTG1,RAB35,Spg20,TM4SF1 |
| Cell Death and Survival,Cell Morphology,Nervous System Development and Function,Neurological Disease,Organ Morphology,Organismal Development,Organismal Injury and Abnormalities,Tissue Morphology | Loss of Purkinje cells | 2.01E-02 |  |  | ABCD2,EI24,OSTM1,PSAP,SMPD1 |
| Cell Cycle,Cellular Assembly and Organization,Cellular Function and Maintenance,DNA Replication, Recombination, and Repair | Duplication of centrosome | 2.03E-02 |  |  | CDK2,NPM1,TTK |
| Amino Acid Metabolism,Lipid Metabolism,Molecular Transport,Small Molecule Biochemistry | Transport of GABA | 2.03E-02 |  |  | SLC6A1,SLC6A11,SLC6A13 |
| Cell Death and Survival,Nervous System Development and Function | Cell survival of neurons | 2.52E-02 |  | -0.447 | PCSK1N,SIGMAR1,SNCA,SOD2,UPF2 |
| Cell Cycle,Cellular Development,Connective Tissue Development and Function | Premature senescence of fibroblasts | 2.52E-02 |  | 0.6 | CDK2,KAT6A,let-7,LIN9,NPM1 |
| Cell Cycle,DNA Replication, Recombination, and Repair | Spindle checkpoint of mitotic cells | 2.52E-02 |  |  | CENPE,IK,TPR,TRIP13,TTK |
| Lipid Metabolism,Small Molecule Biochemistry | Storage of lipid | 2.57E-02 |  | 0.927 | GM2A,NPY,OSTM1,SMPD1 |
| Cell Morphology,Nervous System Development and Function,Neurological Disease,Tissue Morphology | Enlargement of axons | 2.57E-02 |  |  | CNP,CTSV,KCNJ10,OSTM1 |
| Cancer,Endocrine System Disorders,Neurological Disease,Organismal Injury and Abnormalities,Reproductive System Disease | Hyperplasia of pituitary cells | 3.03E-02 |  |  | Cdkn1c,DRD2,PTTG1 |
| Cell Morphology,Cellular Compromise,Nervous System Development and Function,Neurological Disease,Organismal Injury and Abnormalities,Tissue Morphology | Swelling of axons | 3.03E-02 |  |  | CNP,KCNJ10,OSTM1 |
| Cell-mediated Immune Response | Production of natural killer precursor cells | 3.03E-02 |  |  | EOMES,ID2,IKZF5 |
| Cell-To-Cell Signaling and Interaction,Cellular Function and Maintenance,Hematological System Development and Function,Inflammatory Response | Phagocytic capacity of macrophages | 3.03E-02 |  |  | COQ7,let-7,mir-10 |
| Amino Acid Metabolism,Molecular Transport,Small Molecule Biochemistry | Transport of amino acids | 3.23E-02 | Decreased | -2.917 | CRYM,KCNJ10,NTF3,SLC19A1,SLC3A2,SLC6A1,SLC6A11,SLC6A13,SLC7A5 |
| Cellular Function and Maintenance,Nervous System Development and Function | Function of neuroglia | 3.23E-02 |  |  | CCL3L3,DRD2,EGR1,HEPACAM,MAN2C1,Mt2,NDRG2,PSAP,SIGMAR1 |
| Cell Morphology,Cellular Movement,Nervous System Development and Function | Innervation of motor neurons | 3.34E-02 |  |  | EFNB3,FBXO45,RIMS2,TOP2B |
| Cell Morphology,Hematological Disease,Hematological System Development and Function,Hematopoiesis,Lymphoid Tissue Structure and Development,Tissue Morphology | Abnormal morphology of myeloid progenitor cells | 3.63E-02 |  |  | ARID4A,ARID4B,CCND3,CD34,CDK2,Hmgb3,KAT6A,Ly6a (includes others),PLCD1 |
| Cell-To-Cell Signaling and Interaction,Nervous System Development and Function | Synaptic transmission of hippocampal CA1 region | 3.79E-02 |  | 0.152 | CHRM2,EIF4EBP2,GRM5,Nrgn,SLC7A11 |
| Cell Cycle | G1/S phase transition | 3.79E-02 |  | -0.64 | Anp32b,CAMK2G,CDC7,CDK2,CUL1,CUL4A,DBF4,EIF2AK2,GADD45A,GADD45G,ID2,KDR,MCM3,NASP,ORC4,PLK2,PLK3,POLA1,POLE3,PRIM1,RAC1,RCHY1 |
| Cell Morphology,Cell-To-Cell Signaling and Interaction,Nervous System Development and Function | Plasticity of synapse | 3.99E-02 |  |  | ADGRB1,AKAP5,CAMK2G,CDK5,DRD2,EGR1,EIF4EBP2,GRM5,KDR,NCDN,NPAS4,PCDH8,PCP4,PLK2,PLK3,PPT1,RAC1,SNCA,SYNGR1,VGF |
| Carbohydrate Metabolism,Molecular Transport | Quantity of glycogen | 4.25E-02 |  |  | MAN2C1,OSTM1,SLC37A4 |
| Cell-To-Cell Signaling and Interaction,Nervous System Development and Function | Firing of dopaminergic neurons | 4.25E-02 |  |  | ARRB1,CHRNA4,DRD2 |
| Cell Cycle | Endomitosis | 4.25E-02 |  |  | CCND3,CIB1,PLK3 |
| Cardiovascular System Development and Function,Cellular Assembly and Organization,Cellular Development, Skeletal and Muscular System Development and Function | Organization of sarcomere | 4.25E-02 |  |  | MAP2K6,MAP2K7,TXN |
| Embryonic Development,Organismal Development | Arrest in growth of embryo | 4.25E-02 |  |  | CDC7,CDK2,SMARCA5 |
| Cell Morphology,Nervous System Development and Function,Neurological Disease,Tissue Morphology | Abnormal morphology of dopaminergic neurons | 4.48E-02 |  |  | CDK2,CDK5,DRD2,ID2,SNCA,SPR |

*IPA: Ingenuity Pathways Analysis
